# Computational immunohistochemical mapping adds immune context to histological phenotypes in mouse models of colitis

**DOI:** 10.1038/s41598-023-41574-8

**Published:** 2023-09-01

**Authors:** Soma Kobayashi, Christopher Sullivan, Agnieszka B. Bialkowska, Joel H. Saltz, Vincent W. Yang

**Affiliations:** 1 Department of Biomedical Informatics, Renaissance School of Medicine at Stony, Brook University, Stony Brook, NY USA; 2 Department of Medicine, Renaissance School of Medicine at Stony Brook University, Stony Brook, NY USA; 3 Department of Pathology, Renaissance School of Medicine at Stony Brook University, Stony Brook, NY USA; 4 Department of Physiology and Biophysics, Renaissance School of Medicine at Stony, Brook University, Stony Brook, NY USA

**Keywords:** Computational biology and bioinformatics, Immunology, Gastroenterology

## Abstract

Inflammatory bowel disease (IBD) is characterized by chronic, dysregulated inflammation in the gastrointestinal tract. The heterogeneity of IBD is reflected through two major subtypes, Crohn’s Disease (CD) and Ulcerative Colitis (UC). CD and UC differ across symptomatic presentation, histology, immune responses, and treatment. While colitis mouse models have been influential in deciphering IBD pathogenesis, no single model captures the full heterogeneity of clinical disease. The translational capacity of mouse models may be augmented by shifting to multi-mouse model studies that aggregate analysis across various well-controlled phenotypes. Here, we evaluate the value of histology in multi-mouse model characterizations by building upon a previous pipeline that detects histological disease classes in hematoxylin and eosin (H&E)-stained murine colons. Specifically, we map immune marker positivity across serially-sectioned slides to H&E histological classes across the dextran sodium sulfate (DSS) chemical induction model and the intestinal epithelium-specific, inducible *Villin-CreER*^*T2*^*;Klf5*^*fl/fl*^ (*Klf5*^*ΔIND*^) genetic model. In this study, we construct the beginning frameworks to define H&E-patch-based immunophenotypes based on IHC-H&E mappings.

## Introduction

Crohn’s disease (CD) and Ulcerative Colitis (UC) are the two major subtypes of Inflammatory Bowel Disease (IBD). IBD is a state of chronic, dysregulated inflammation of the gastrointestinal tract. Approximately 1.6 million Americans are afflicted by IBD, and as many as 70,000 new cases are diagnosed yearly^1^. Time-trend analyses of population-based IBD studies from the mid-1900s to 2000s showed that 75% of CD and 60% of UC studies showed significant increases in disease incidence rates^2^. Thus, the prevalence of IBD is expected to grow, resulting in more considerable healthcare cost burdens.

The most common symptoms are abdominal pain, diarrhea, nausea, vomiting, and rectal bleeding^1,3^. While an intestinal disease, IBD also has extraintestinal manifestations. These include arthritis, ankylosing spondylitis, erythema nodosum, iritis, uveitis, and primary sclerosing spondylitis^3–5^. CD can also increase the risk for cancer, pulmonary, gastrointestinal, and urinary tract disease^1,3^, while UC is associated with advanced colorectal cancer risk^1,6^. A significant difference between CD and UC is in the disease pattern of intestinal involvement. CD is discontinuous and can occur anywhere along the gastrointestinal tract, while UC has a continuous pattern involving only the colon^7^. Symptomatically, this translates to visibly bloody stool in UC and fecal occult blood in CD^7^.

As with other immune diseases, researchers have characterized CD and UC by the dominant CD4 + T-helper subset during inflammation. Classically, CD is considered a T-helper type 1 (Th1)-mediated disease and UC T-helper type 2 (Th2)-mediated. However, the role of the T-helper type 17 (Th17) cells is also now being recognized in both^8^. Clinically, IBD is treated as an immune disease with monoclonal antibodies targeting cytokines produced by T-helper populations implicated in IBD^9^. For CD, anti-TNFα therapies (infliximab, adalimumab) are first-line^9^. However, other biologics, such as anti-α_4_β_7_ (vedolizumab) and anti-IL-12/IL2-3 (ustekinumab), have shown efficacy as well^9^. IL-12 and IL-23 are cytokines required to maintain the Th1 and Th17 populations, respectively, while the α_4_β_7_ integrin is necessary for adaptive T cells to home to the intestine^10^. Despite the growing range of biologics, patients are not characterized for therapy decisions. In UNITI, a clinical trial of moderate to severe CD patients who were non-responders to first-line anti-TNFα or corticosteroids, ustekinumab showed a significant benefit in clinical response and remission^11^. There may have thus been patients who would have benefited from earlier ustekinumab. As such, pathogenesis and immune responses in IBD must be better understood to stratify patients and improve the efficiency of treatment decisions. To this end, researchers have heavily studied mouse models to further elucidate disease pathogenesis and identify new treatment targets.

Mouse models play an essential role in the study of human disease^12–15^. The power of mouse models lies in the capacity to reproduce specific disease phenotypes consistently and reliably across experiments. This has encouraged scientific growth as research groups worldwide can study phenotypes to disseminate results and collaborate. Depending on the mouse model, disease induction can occur in various ways, such as chemical insults or genetic modifications. To isolate and attribute experimental outcomes to specific independent variables, mice are typically age-, gender- and genetically-matched. Highly controlled experiments are integral in mechanistic studies dissecting the pathophysiology of disease processes but are also a double-edged sword. Pathways in mice can be studied comprehensively, but direct translation to the clinic often does not result. This is likely due to the high heterogeneity of patient populations across factors controlled in mice, like age, gender, and genetics. Additionally, there are vast ranges in patients’ social behaviors and comorbidities.

Acknowledging the shortcomings of mouse models should not be a dismissal of their value in academic research. Instead, the shortcomings should be addressed to fully expand the potential of preclinical mouse modeling in translation to human disease. One approach is to standardize multi-mouse model studies. Individual mouse models simulate specific phenotypes observed in clinical syndromes and should be aggregated for a more comprehensive and heterogeneous phenotypic pool within experiments. In the case of IBD, mouse models exhibit colitis but vary in flavors of the immune response, detected histological features, survival, and treatment response^12,16^. While more mouse models lead to more data and analytical load, computational advances in processing power, parallel processing, concepts, and memory^17–19^ open the doors to these approaches. Genomic and histological modalities have significantly benefited from these new capabilities. Here, we developed a computational pipeline to assess histopathology across two mouse models of colitis and controls.

Specifically, we generated a dataset of IHC-H&E stacks. In previous work, we trained a ResNet-34 model to detect regions ‘Involved’ and ‘Uninvolved’ with disease in H&E-stained colons from controls and three mouse models of colitis^20^. We identified four diseased histology classes—“Inflammatory” (mild influx of immune cells), “Crypt Dropout” (crypt epithelium loss), “Crypt Dilation” (crypt lumen expansion), and “Distorted Glands” (architectural distortion). To build upon this H&E pipeline, we took serial sections with logged order from formalin-fixed paraffin-embedded murine colons. We utilized the dextran sodium sulfate (DSS) colitis model and the *Villin-CreER*^*T2*^*;Klf5*^*fl/fl*^ (*Klf5*^*ΔIND*^) model, wherein inducible intestinal epithelium-specific knockout of *Klf5* disrupts barrier function and causes colitis. For each IHC-HE stack, H&E and IHC staining for CD8b, CD3, and CD4 were performed in the same order. IHC marker positivity was computationally quantified and mapped back to H&E patch classes and ‘Involved’ and ‘Uninvolved’ regions detected by our previous pipeline. We found that the ‘Crypt Dilation’ and ‘Crypt Dropout’ patch classes in DSS-treated mice are characterized by CD8b positivity depletion, while the ‘Crypt Dilation’ patch classes in the *Klf5*^*ΔIND*^ model maintained CD8b positivity. Conversely, the DSS-treated mice exhibited the highest CD8b positivity in ‘Uninvolved’ regions, indicating that this model may hold value in studying protective CD8b + populations. Our study explores the potential of IHC-H&E mappings to build upon H&E histological classes with immune context with an eye toward the future incorporation of additional IHC markers.

## Results

### Mouse cohort

We previously developed a ResNet-34-based^21^ pipeline that detects and characterizes regions involved or uninvolved with disease in hematoxylin and eosin (H&E)-stained colons across multiple mouse models of colitis on a patch basis (‘Involved’ versus ‘Uninvolved’ Classifier)^20^. The ‘Involved’ versus ‘Uninvolved’ Classifier pipeline identified five ‘Involved’ H&E patch classes: ‘Inflammatory’ (Milder, more heterogeneous phenotype with immune cell nuclei influx), ‘Crypt Dropout’ (loss of epithelium with displacement by stroma and immune cells), ‘Crypt Dilation’ (crypt lumen space expansion), and ‘Distorted Glands’ (crypt structure distortion)^20^. These ‘Involved’ patch classes were present in significantly different proportions across our mouse models. Furthermore, per-mouse proportions of ‘Uninvolved’ patches and ‘Involved’ patch classes along the colon were sufficient to train a linear determinant analysis (LDA) classifier to predict mouse model. In other words, detecting and quantifying these patch classes was informative in histologically distinguishing between our mouse models.

Given the value of these patch classes in classifying between colitis mouse models, the goal of this study was to build upon the H&E pipeline by adding the capacity to incorporate immunohistochemically (IHC)-generated immune context (Supplementary Fig. 1a). To explore developing an improved IHC-H&E pipeline, two of the colitis mouse models from our previous study were included, and the mouse treatment schedules are shown in Supplementary Fig. 1b. The first colitis mouse model is the genetic *Villin-CreER*^*T2*^*;Klf5*^*fl/fl*^ (*Klf5*^*ΔIND*^) model, wherein an inducible intestinal epithelium-specific knockout of *Klf5* causes colitis^22,23^. KLF5 is a pro-proliferative zinc finger transcription factor essential for maintaining intestinal epithelial barrier function. KLF5 is critical in the regeneration, proliferation, and replenishment of mature, differentiated cells in the intestinal epithelium^24–27^. As heterogeneous cell populations of the highly dynamic intestinal epithelium turn over and must be replaced every 3–5 days, stem-cell-driven replenishment of the epithelial barrier is crucial. Inducible intestinal-epithelium specific deletion of KLF5 impairs this replenishment to cause impaired epithelial barrier function and Th17-dependent colitis with pathological features and microbiotic changes reflective of human IBD^23^. The ‘Involved’ versus ‘Uninvolved’ classifier pipeline previously revealed that 5T-*Klf5*^*∆IND*^ mice are enriched in ‘Inflammatory’ and ‘Crypt Dilation’ patch classes ^20^.

The second colitis mouse model is the chemical dextran sodium sulfate (DSS) model (Supplementary Fig. 1b)^28–30^. While the mechanism of DSS-induced colitis has not been fully elucidated, induction is believed to depend on tissue penetrance of the chemical to allow disruption of the intestinal epithelial monolayer and barrier integrity^31^. Although Th1 cells have been implicated in DSS colitis, evidence suggests it is a myeloid-mediated model. For example, DSS-induced colitis still occurs in immunocompromised SCID^32^ and *RAG*^*-/-*^ mice^28,33^, which cannot generate fully mature adaptive immune responses. However, others have reported that while the development of colitis with a high DSS dose (5% in drinking water) was comparable between Bl/6 control and *RAG1*^*-/-*^ mice, progression was less severe in *RAG1*^*-/-*^ mice with a lower DSS dose (1.5% in drinking water)^34^. The contribution of lymphocytic populations may thus be more appreciable in the lower 2.5% setting used in this study than the more severe 5%, as was utilized by the studies in^32^ and^33^. However, it is essential to note the significance of the innate immune response in human IBD. Clinically, neutrophils are one of the first recruited populations^35^ and the only marker of “active” inflammation in the intestine. Cryptitis (neutrophil infiltration within the epithelium of crypts) and crypt abscesses (neutrophil accumulation in crypt lumens) are both observed in IBD^36–38^. Furthermore, first-line biologic therapies for IBD target TNFα^9^, produced by Th1 cells and innate populations. As such, a mouse model with components of myeloid-mediated pathogenesis holds potential value in deciphering clinical disease, especially when paired with other more adaptive-mediated mouse models. In this study, colons were collected from 16 total mice, covering 5 5T-*Klf5*^*∆IND*^, 4 DSS-treated, and 7 control mice (Table 1).

**Table 1:** Mouse cohort overview.

	Genotype	Injection	# Days injection	DSS/H_2_O	# Days DSS/H_2_O	Abbrieviation	Induction status	Comparison group	Number
Stanined for H&E and IHC: CD3, CD4, CD8b	*Klf5*^*∆IND*^	CO	5	N/A	0	5C-*Klf5*^*∆IND*^	Control	Control	4
	*Klf5*^*∆IND/*+^	N/A	0	0	0	*Klf5*^*∆IND/*+^	Control	Control	2
	*Klf5*^*WT*^	N/A	0	H_2_O	7	*Klf5*^*WT*^ + H_2_O	Control	Control	1
	*Klf5*^*∆IND*^	TAM	5	N/A	0	5 T-*Klf5*^*∆IND*^	Colitis	5 T-*Klf5*^*∆IND*^	5
	*Klf5*^*∆IND/*+^	CO	5	DSS	7	5C-*Klf5*^*∆IND/*+^ + DSS	Colitis	DSS	1
	*Klf5*^*WT*^	N/A	0	DSS	7	*Klf5*^*WT*^ + DSS	Colitis	DSS	3

### Computational registration of serially-sectioned slides

**Figure 1:**
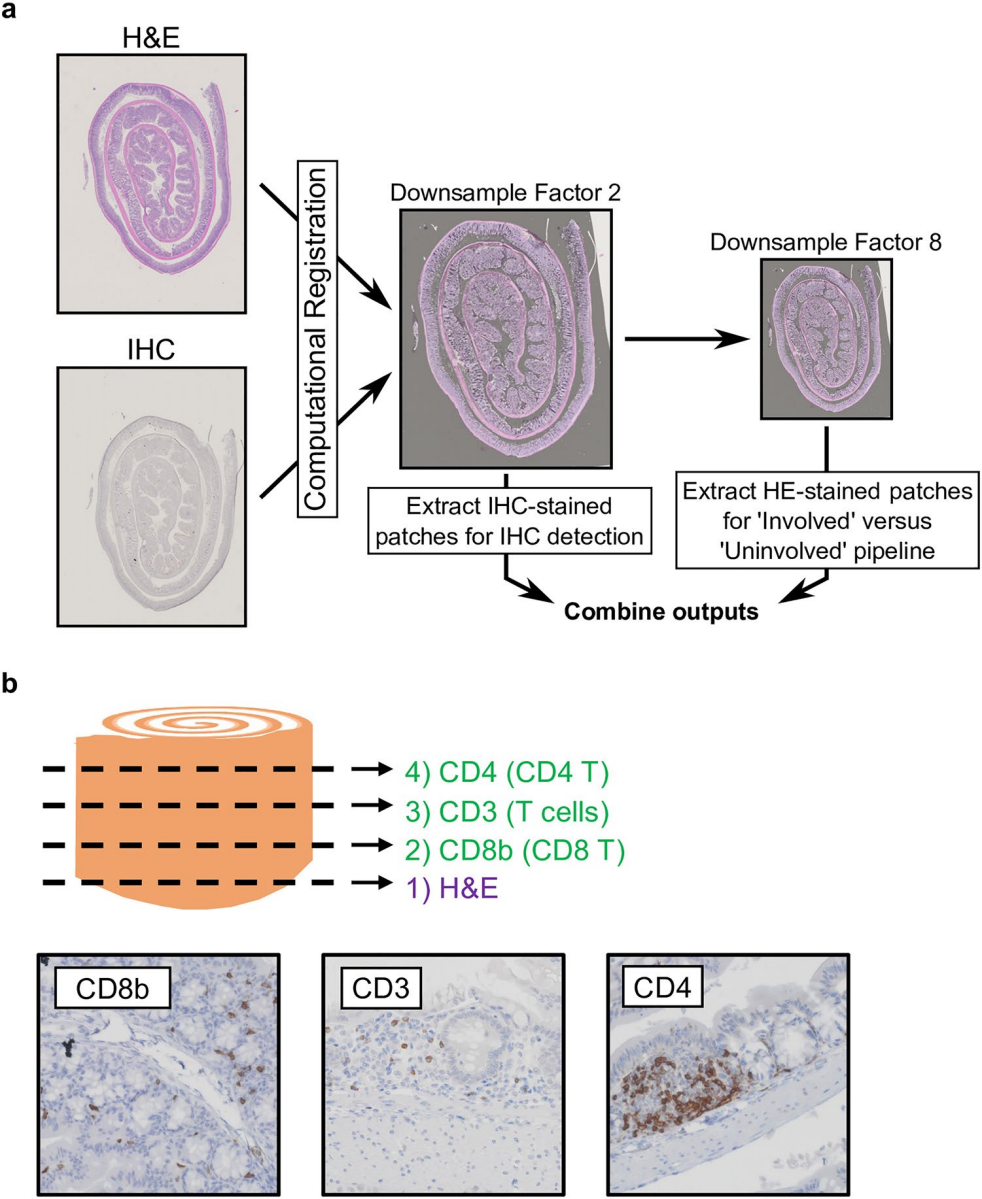
IHC-HE stack overview. (**a**) An overview of the analysis approach is provided. After downsampling by a factor of 2, single-marker immunohistochemistry (IHC)-stained and hematoxylin-and-eosin (H&E)-stained whole slide images (WSIs) computationally registered using WSIReg2D ^40^. IHC detection is performed at this resolution. The H&E WSI must be downsampled by an additional factor of 4 so extracted image patches match the required resolution for the ‘Involved’ versus ‘Uninvolved’ Classifier ^20^. IHC and H&E outputs are then combined. (**b**) Graphic showing order in which stains occur for all samples with example IHC-stained patches.

The general approach for combining IHC and H&E is shown in Fig. 1a. All histological slides are scanned at 40X resolution into whole-slide images (WSIs), which are digitized gigapixel-size image files that allow for subsequent computational analysis. Computational registration occurs at a higher resolution (downsample factor of 2). The H&E WSIs are then downsampled by another factor of 4 before patches are extracted and fed into the ‘Involved’ versus’ Uninvolved’ Classifier pipeline. As formalin-fixed paraffin-embedded (FFPE) swiss-rolled^39^ colons are serially sectioned with logged ordering, slides are stained in the same order for all samples. The first section was stained for H&E. Single-marker immunohistochemistry (IHC) staining for CD8b (CD8b + T cells), CD3 (general T cells), and CD4 (CD4 + T cells, some myeloid populations) was then performed for slides 2, 3, and 4, respectively (Fig. 1b).

**Figure 2:**
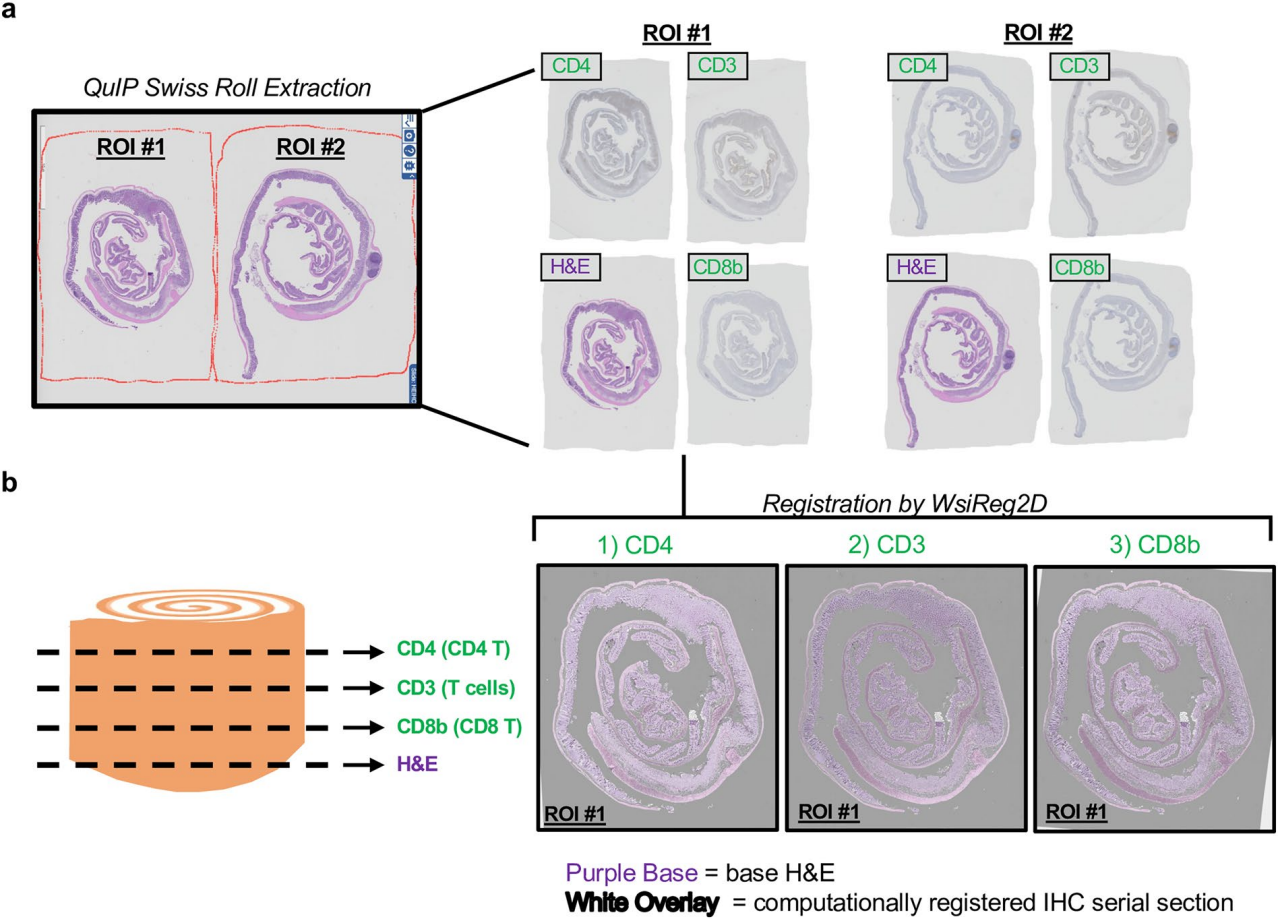
Individual swiss roll extraction and registration. (**a**) Example of manual annotations from QuIP ^40^ with corresponding, extracted swiss rolls from along IHC-HE stack. (**b**) Tissue masks of registered, IHC-stained swiss rolls are overlayed onto base H&E-stained swiss roll from slide 1. High overlap between white tissue mask and H&E-stained tissue indicates adequate registration quality. Computational registration was performed using WSIReg2D ^40^.

The collected serial sections are ordered and close in proximity and should have similar appearing morphology. However, when digitized by the scanner, their general position and orientation will be inconsistent, as they are still sectioned onto glass slides separately (Fig. 2a). As such, the WSIReg2D package^40^ was applied to perform deformable, non-linear registrations of these serially-sectioned WSIs. The goal of computational registration is to align WSIs so that corresponding colonic regions will be present when traversing along the vertical z-axis direction of the stacks. In other words, after examining a portion of tissue in one WSI, the same XY coordinates can extract corresponding tissue regions from other WSIs in the stack. Since these are serially-sectioned slides, registration is performed iteratively. For example, slide two is first registered onto the H&E-stained slide one. Slide three is then registered onto the already registered slide two, and so on. This process ensures that each section is registered onto the slide of closest anatomic proximity.

However, when collecting samples, colons are swiss-rolled and then cut in half before formalin fixation and paraffin embedding, so WSIs contain two swiss tissue sections each. WSIs are uploaded onto QuIP, a web-based WSI viewer maintained by the Stony Brook Department of Biomedical Informatics^41^. For each WSI, individual swiss rolls were outlined with manual annotations converted into polygon coordinates (Fig. 2a). Swiss rolls were cropped along polygon coordinates, then pasted onto a rectangular, white background.

The iterative registration process was performed on these extracted, individual swiss rolls. For each IHC-stained WSI, tissue masks of the registered swiss rolls were generated and overlayed over the base H&E-stained swiss rolls. Overlap of white IHC-stained tissue masks with purple, H&E-stained swiss rolls indicates proper registration of serially-sectioned WSIs (Fig. 2b). While both swiss rolls per mouse are kept throughout the pipeline, only one per mouse is included for the final IHC detection analyses based on registration quality.

### Patch extraction from H&E- and IHC-stained, registered WSIs

The H&E-stained WSIs are downsampled by an additional factor of 4 to match the resolution required by our ‘Involved’ versus ‘Uninvolved’ Classifier^20^. As with previous work, the Small Patch Classifier that detects ‘Tissue’, ‘Submucosa’, ‘Muscle’, and ‘Background’ regions from tiny 32 × 32 pixel patches was applied^20^. Upon extraction of 32 × 32 pixel patches and application of the Small Patch Classifier, tissue maps are generated for each sample, capturing regions of interest (‘Tissue’ and ‘Submucosa’) along with non-informative regions (‘Muscle’ and ‘Background’) (Supplementary Fig. 2a). For every 224 × 224 pixel patch extracted for H&E analysis, the corresponding region is inspected within tissue maps to determine if enough areas of interest exist. Practically, a 65% cutoff is implemented. If more than 65% area of a patch is deemed non-informative, it is not kept (Supplementary Fig. 2b). Kept H&E patch coordinates are then utilized to guide IHC patch extraction (Supplementary Methods).

### H&E classifications

The first step was to apply the ‘Involved’ versus ‘Uninvolved’ H&E pipeline^20^ to the H&E samples, and we briefly describe the approach. The ‘Involved’ versus ‘Uninvolved’ Classifier can detect regions involved and uninvolved with disease from H&E-stained patches. However, since a regular grid simply tiles the WSIs to extract patches, classification bias may occur in some situations based on what was present in a patch field of view. For example, how much tissue is in a patch or the proportion of tissue in a patch of abnormal histology may affect ‘Involved’ versus ‘Uninvolved’ predictions. Relatedly, linear translations of the WSIs in any direction would lead to extracting a different set of patches in terms of image content. To this end, overlapping patch extraction is performed to reduce any classification dependence on what image patch happened to be extracted from a tissue region.

**Figure 3:**
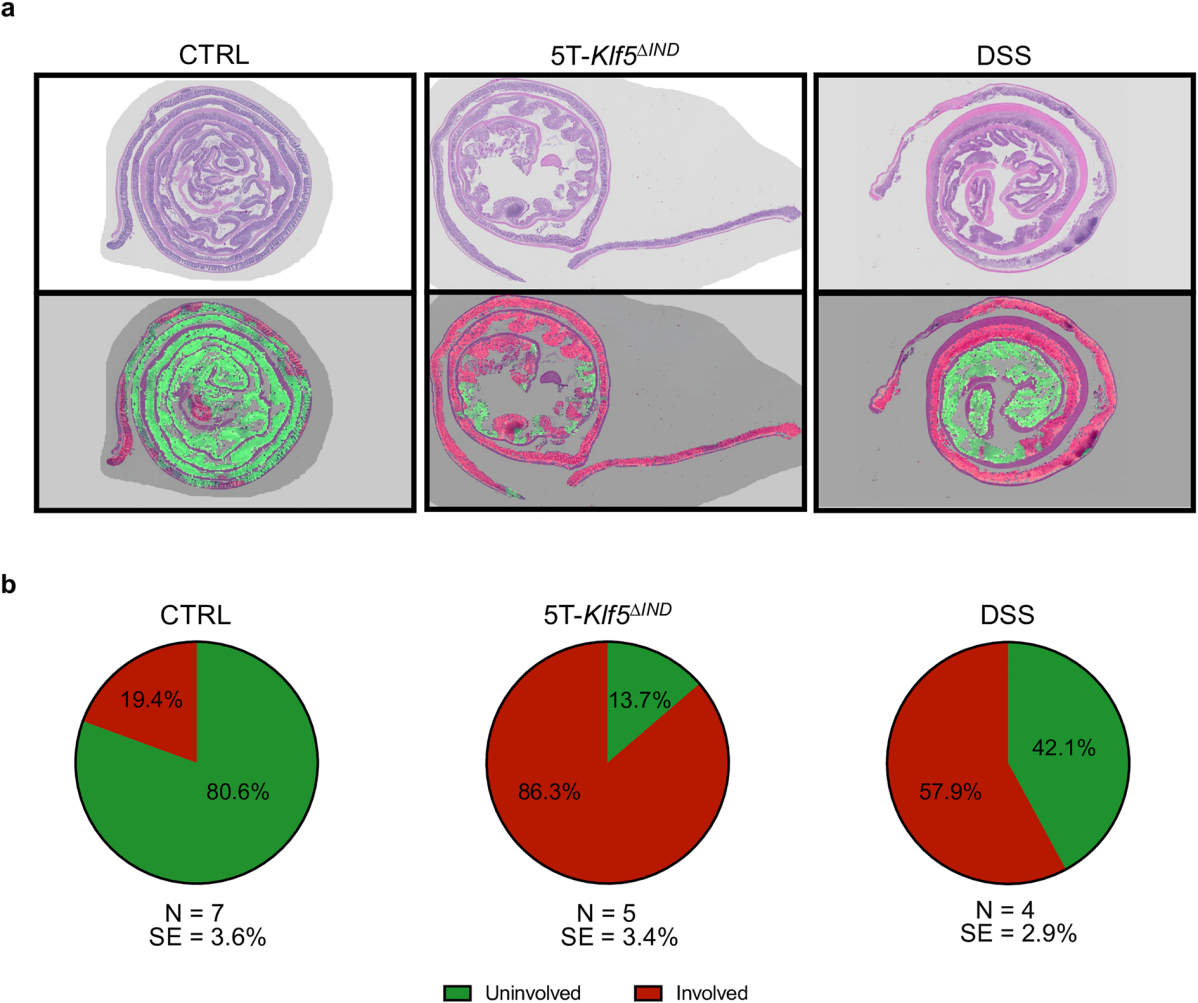
Binary ‘Involved’ versus ‘Uninvolved’ Classifier outputs. (**a**) Representative input H&E-stained swiss rolls are shown with corresponding ‘Involved’ (red) versus ‘Uninvolved’ (green) prediction overlays. (**b**) Total proportion of ‘Involved’ and ‘Uninvolved’ patches for each condition across all samples in dataset.

The initially kept H&E-stained patches were utilized as landmarks to direct extraction of overlapping patches to incorporate more spatial context into patch predictions (Supplementary Methods). The ‘Involved’ versus ‘Uninvolved’ classifier is applied to each overlapping patch, and prediction confidences are averaged at every pixel in the WSI. As with previous work, overlays showing areas of at least 50% confidence of ‘Involved’ and ‘Uninvolved’ predictions in green and red over input H&E are generated (Fig. 3a). Any regions with ‘Background’ and ‘Muscle’ classifications from the tissue maps were eliminated from these overlays. ‘Involved’ and ‘Uninvolved’ patch proportions were quantified across mouse conditions and indicated that, while control mice show some ‘Involved’ predictions, they occur at low frequency (Fig. 3b). Provided no colitis induction occurred in these mice, these are likely patches with architectural distortion during tissue collection and preparation. Per mouse proportions are available in Supplementary Fig. 3.

### IHC marker positivity detection

During IHC staining, hydrogen peroxidase-conjugated antibodies are bound only where the desired IHC marker target is present. These IHC slides are then developed with 3,3’-Diaminobenzidine (DAB), a substrate that converts into a brown chromogen in hydrogen peroxidase. Thus, the brown stain color represents areas of IHC marker target presence in these samples (Fig. 1b). There is, therefore, a need to detect regions within image patches where brown color occurs and quantify how much marker was present.

This study adopted the Scikit-image rgb2hed function that takes input IHC-stained RGB images and extracts a DAB channel^42^. IHC detection is performed on a patch-by-patch basis on initially-extracted patches, and a DAB channel image is extracted from each (Supplementary Fig. 4). Using the Pillow library^43^, DAB channel images were converted to grayscale, contrast enhanced, then thresholded to generate positive DAB detection masks (Supplementary Fig. 4). Connected objects were then detected from the positive DAB detection masks. To reduce noise and non-specific staining, size thresholds were implemented to exclude connected objects above or below selected size cutoffs. While our approach detected brown DAB staining, dark black hematoxylin crystal artifacts were also being detected as false positives. To address this issue, the original IHC-stained RGB patches are color inverted. In this inverted color space, connected objects corresponding to the crystal artifacts can be selected by intensity thresholding (Supplementary Fig. 4). Finally, the remaining connected objects excluding hematoxylin crystal artifacts are counted to quantify presence of lymphocytes with marker positivity. Notably, lymphoid aggregate regions are excluded from IHC detection, as these areas typically have high lymphocyte-marker positivity regardless of the local disease phenotype.

While a more straightforward image processing approach, an advantage is that a new detection model does not need to be explicitly trained for each marker. However, while all slides were developed with the same DAB substrate, intensity thresholds, contrast enhancement scales, minimum and maximum size cutoffs must be optimized separately for each marker. These values can depend upon IHC marker-specific factors like the morphology of stained cells, staining patterns, or intensity. It should also be noted that there are limitations to this approach. For example, clumped cells cannot be segmented into individual cells and may be interpreted as one connected object. Additionally, detection threshold parameters were kept stringent to reduce non-specific stain detection and focus on the strongest DAB signals. However, this approach reliably detects differences between regions with high, medium, and low IHC marker positivity (Supplementary Fig. 5), which should capture more significant shifts of IHC-defined immune contexts between H&E patch classes and mouse models.

### “Pseudo-multiplex IHC” overlay generation

**Figure 4:**
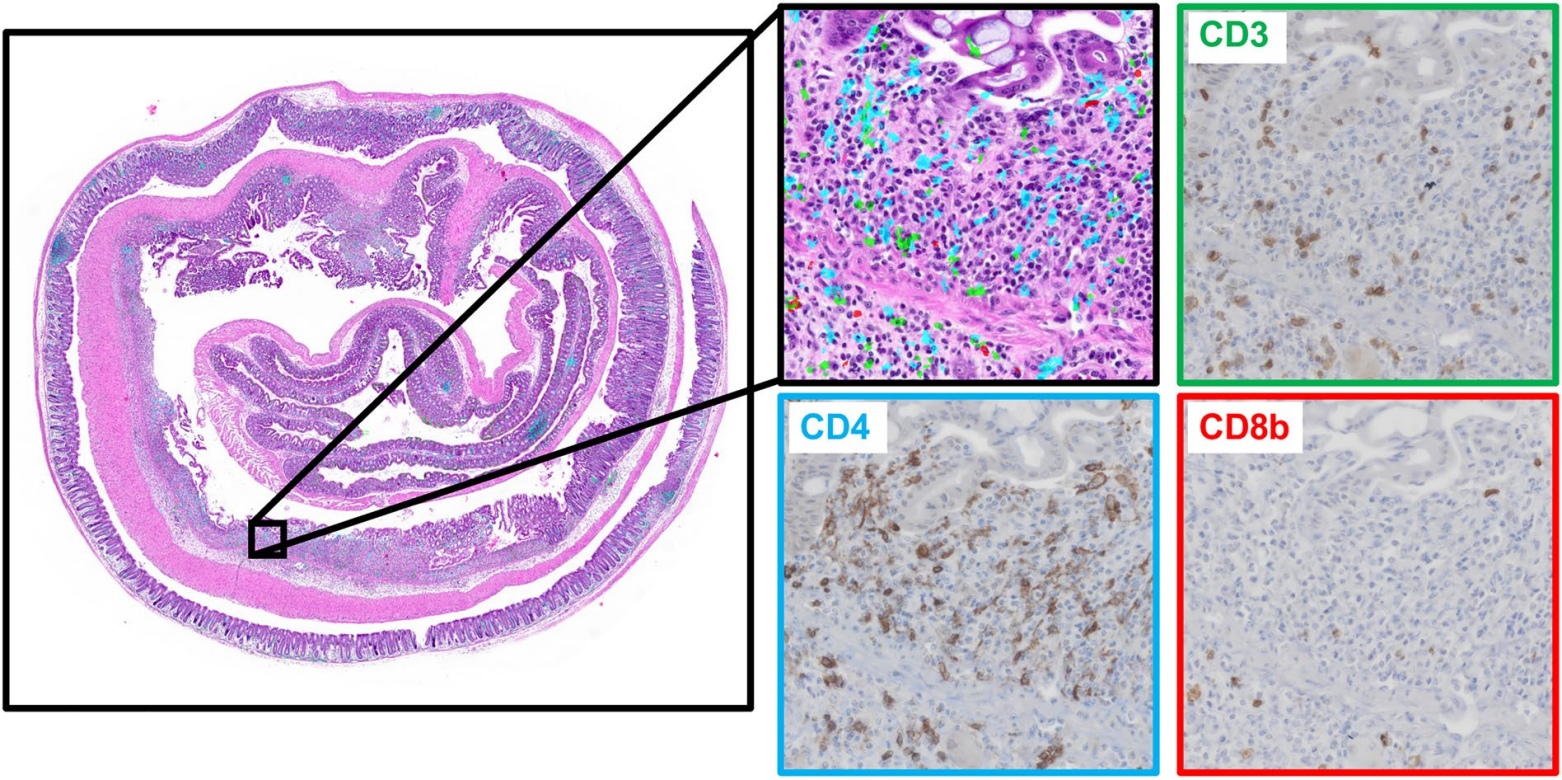
Example “pseudo-multiplex” IHC. Detected IHC marker positivity from serially sectioned slides were overlayed onto the base H&E-stained WSI (slide position 1) after computational registration. An example “pseudo-multiplex” IHC patch with corresponding patches from serially-sectioned, IHC-stained WSIs is provided.

As the serially-sectioned slides are computationally registered and IHC marker positivity detected, IHC detection results can be overlayed onto the H&E slides. IHC marker detection maps were collected across all IHC-stained WSIs along a mouse’s IHC-HE stack via patch XY coordinates on a patch-by-patch basis. The IHC detection tissue masks are overlayed onto base H&E-stained WSIs, and each IHC marker is provided a different color to generate “pseudo-multiplex IHC” outputs (Fig. 4). Additionally, specific regions of pseudo-multiplex IHC outputs can be examined along with corresponding patch regions from each IHC-stained WSI.

### IHC-HE mapping shows shifts in immune marker prevalence between ‘involved’ and ‘uninvolved’ regions across 5T-Klf5^∆IND^ and DSS-treated mice

**Figure 5:**
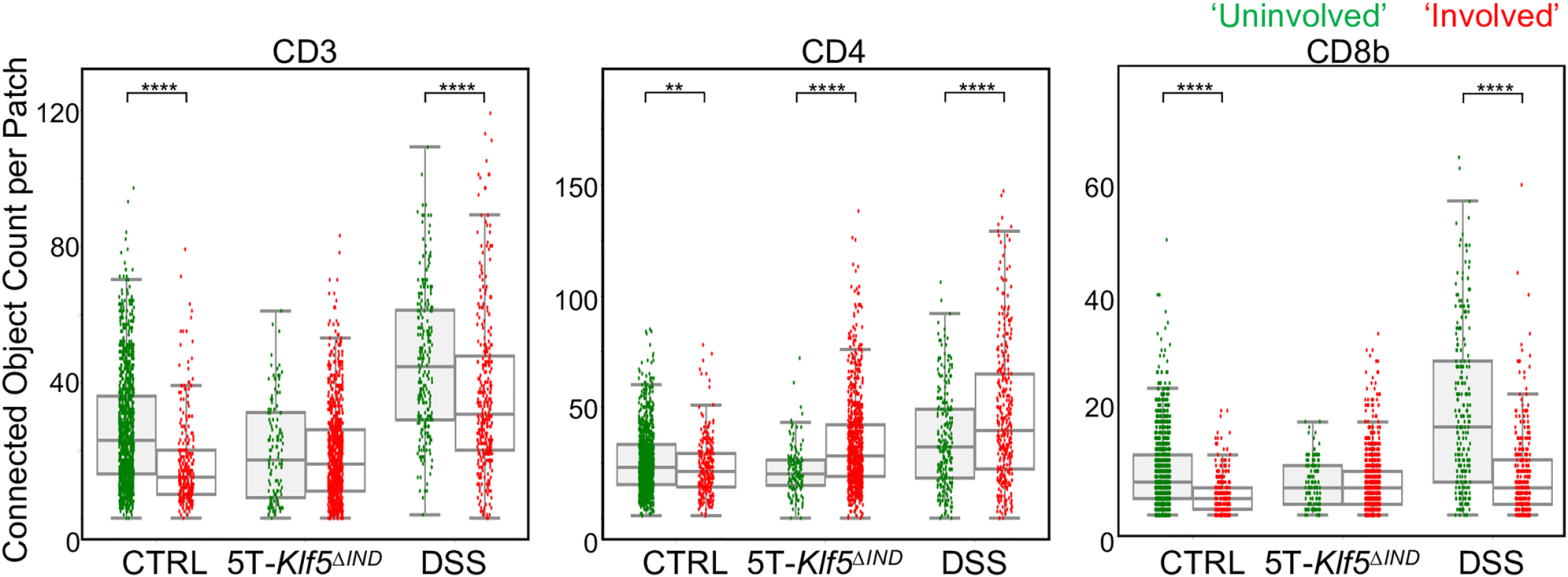
IHC markers show shifts between ‘Uninvolved’ and ‘Involved’ regions across mouse models. IHC connected object counts were compared between ‘Uninvolved’ and ‘Involved’ regions for each mouse condition. Image patches comparing lymphoid aggregates were excluded from analysis. Student’s t-test was performed between ‘Uninvolved’ and ‘Involved’ patches for each mouse condition with *p < 0.05, **p < 0.01, ***p < 0.001, ****p < 0.0001.

IHC markers were next quantified across ‘Involved’ patch classes defined on H&E. Initially, detected IHC connect object counts were compared between ‘Uninvolved’ and ‘Involved’ regions for our mouse models (Fig. 5). Per-mouse data is available in Supplementary Fig. 6. The 5T-*Klf5*^*∆IND*^ mice showed enrichment for CD4 positivity in ‘Involved’ relative to ‘Uninvolved’ regions but not for CD3. Given the Th17-mediated colitis phenotype of the 5T-*Klf5*^*∆IND*^ model, this may reflect a shift in the composition of CD3 + populations but not the overall count. An alternative possibility is protective CD3 + population recruitment in the ‘Uninvolved’ regions. The DSS-treated mice showed significant decreases in CD3 and CD8b positivity in ‘Involved’ regions relative to ‘Uninvolved’, and significant increases in ‘Involved’ region CD4 positivity. This may reflect the contribution of myeloid cells in the DSS model, as some of these populations can also express CD4. Relative to CD3 + or CD8b + cells. CD4 + cells have more morphologic heterogeneity than CD3 + or CD8b + cells (Fig. 1b, Supplementary Fig. 5). Finally, there is no significant elevation of immune marker positivity in ‘Involved’ regions relative to ‘Uninvolved’ regions of control mice, indicating that these ‘Involved’ predictions are likely related to architectural distortion from swiss roll collection and do not represent areas of inflammation. Colons may have experienced slight damage during the harvesting protocol garnering ‘Involved’ predictions.

### IHC-HE mapping shows difference in enrichment of marker positivity in ‘involved’ k-means patch classes across 5T-Klf5^∆IND^ and DSS-treated mice

**Figure 6:**
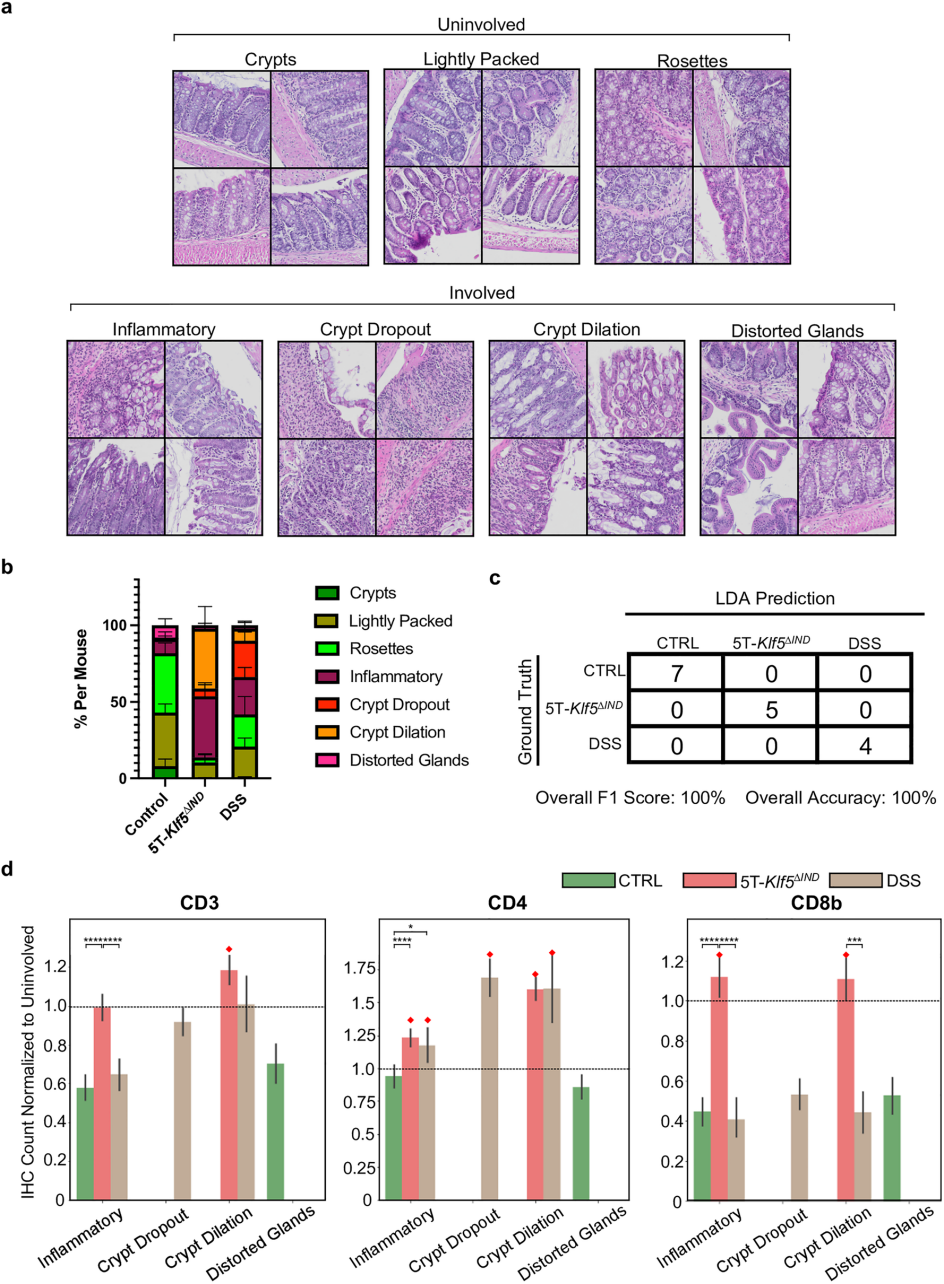
IHC mapping to H&E ‘Involved’ k-means patch classes. (**a**) Example image patches from across the dataset for each of the ‘Uninvolved’ and ‘Involved’ k-means patch classes. Patches most representative of class labels are shown. (**b**) Stacked bar plot showing proportion of patch classes along swiss rolls across mouse conditions. K-means patch class proportions for every sample were generated before plotting. (**c**) Linear determinant analysis classifier from ^20^ utilized ‘Involved’ k-means patch class and ‘Uninvolved’ patch proportions to predict mouse models from H&E-stained swiss rolls. (**d**) IHC connected object counts for ‘Involved’ patches are normalized within each mouse by that mouse’s average connected object count across all ‘Uninvolved’ patches. In addition, analysis is focused on ‘Involved’ patch classes present in at least 10% of ‘Involved’ patches for a specific mouse condition. Dashed lines represent 1.0 cutoffs for normalized IHC counts to indicate enrichment. Red diamonds indicate any findings above 1.0 mark. One-way ANOVA was performed across normalized IHC positivity from patches with *p < 0.05, **p < 0.01, ***p < 0.001, ****p < 0.0001.

While the ‘Involved’ and ‘Uninvolved’ classifications identify regions with and without histological abnormalities within murine colons, histological characterizations need to move beyond these binary classifications to distinguish between mouse models. To this end, the final portion of the ‘Involved’ versus ‘Uninvolved’ Classifier pipeline was applied to the samples to quantify previously discovered patch classes of H&E histology^20^. As previously described, feature extraction and clustering were performed separately on ‘Involved’ and ‘Uninvolved’ patches. This categorized ‘Involved’ H&E patches into one of the ‘Inflammatory’ (Milder, more heterogeneous phenotype with an influx of immune cell nuclei), ‘Crypt Dropout’ (loss of epithelium and displacement by stroma and immune cells), ‘Crypt Dilation’ (expansion of crypt lumen spaces), or ‘Distorted Glands’ (distortion of crypt structures) classes (Fig. 6a). ‘Uninvolved’ patches are classified into ‘‘Crypts’ (test-tube-like perspective of crypt structures), ‘Lightly Packed’ (more space between crypts often accompanied by immune cell nuclei), and ‘Rosettes’ (Ring-like cross-section perspective of crypts).

For each swiss roll, proportions of ‘Involved’ and ‘Uninvolved’ k-means patch classes were calculated (Fig. 6b). Per-mouse proportions are available in Supplementary Fig. 7. As with previous work, ‘Involved’ k-means patch class and ‘Uninvolved’ patch proportions were fed into the pipeline’s LDA classifier as an internal control to confirm that these patch class proportions successfully predict whether the specimen came from either of the mouse models or control (Fig. 6c). The goal is to build upon the previous pipeline’s k-means patch class outputs by adding an IHC-defined immune context. Notably, the LDA classifier still properly makes control predictions for control mice, again indicating that the ‘Involved’ predictions for these samples were likely architectural distortions during tissue collection.

IHC marker positivity was mapped across H&E k-means ‘Involved’ patch classes. For every mouse, the average IHC-connected object count across all ‘Uninvolved’ patches was first calculated for each IHC marker. Next, for every ‘Involved’ patch from a mouse, IHC-connected object counts were normalized by the average ‘Uninvolved’ IHC count for that mouse. This approach allowed the identification of IHC markers enriched in ‘Involved’ patch classes relative to ‘Uninvolved’ regions within each mouse. Finally, not all ‘Involved’ patch classes are in appreciable quantities across mouse models (Supplementary Table 1). As a result, incorrect k-means patch class predictions, especially at low frequencies per mouse model, can skew results as connected object counts are not currently normalized to patch frequency. Therefore, to explore the potential of IHC-HE mappings to define histological phenotypes, the current analysis was directed toward k-means patch classes in at least 10% of ‘Involved’ patches for a mouse model across the entire dataset (Fig. 6d, Supplementary Table 1). Any low-frequency patch classes are provided a weight of 0 (ex: ‘Distorted Glands’ in either 5T-*Klf5*^*∆IND*^ or DSS-treated mice).

**Figure 7:**
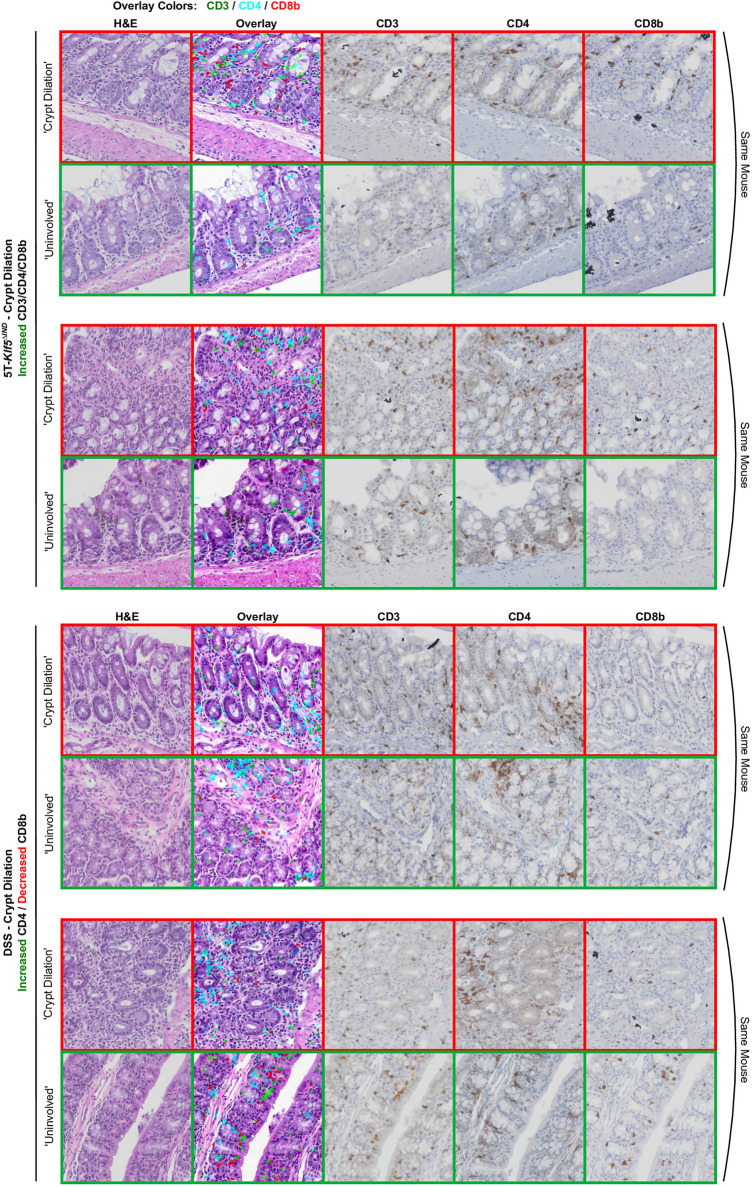
Example ‘Crypt Dilation’ patches from 5T-*Klf5*^*∆IND*^ and DSS-treated mice. An H&E-stained, “pseudo-multiplex” IHC, CD3-stained, CD4-stained, and CD8b-stained patch is shown for each tissue region. For every ‘Crypt Dilation’ patch, a paired ‘Uninvolved’ region from that same mouse is shown. ‘Crypt Dilation’ patches in 5T-*Klf5*^*∆IND*^ mice show an enrichment of CD8b marker positivity, while DSS-treated mice show a depletion.

The 5T-*Klf5*^*∆IND*^ mice were previously found to be enriched in the ‘Inflammatory’ and ‘Crypt Dilation’ classes^20^. The ‘Inflammatory’ patches from 5T-*Klf5*^*∆IND*^ mice have higher CD4 and CD8b marker positivity relative to ‘Uninvolved’ regions within the same mice (Fig. 6d). These normalized IHC counts are significantly higher than DSS-treated and control mice counterparts. Additionally, while normalized CD3 positivity in ‘Inflammatory’ patches was not enriched relative to ‘Uninvolved’ patches (normalized count of around 1.0), they are significantly elevated with respect to Control and DSS-treated mice. The 5T-*Klf5*^*∆IND*^ ‘Crypt Dilation’ patches were enriched for all three IHC markers relative to ‘Uninvolved’ regions from the same mouse. However, these patch classes' normalized CD8b count was above 1.0 and significantly elevated relative to control and DSS-treated mice. Interestingly, the ‘Crypt Dilation’ patches from DSS-treated mice show a similar IHC marker profile besides a depletion in normalized CD8b count. Therefore, H&E patches and their corresponding IHC-stained patches can be extracted and examined to confirm the lack of CD8b staining in DSS-treated Crypt Dilation patches (Fig. 7). Qualitatively, while the DSS ‘Crypt Dilation’ patches have expanded and dilated gland lumens, the glands are more spaced. Therefore, while the glandular morphologies and lumen expansion may be common features for ‘Crypt Dilation’ patches across these mouse models, the associated immune microenvironment may differ.

**Figure 8:**
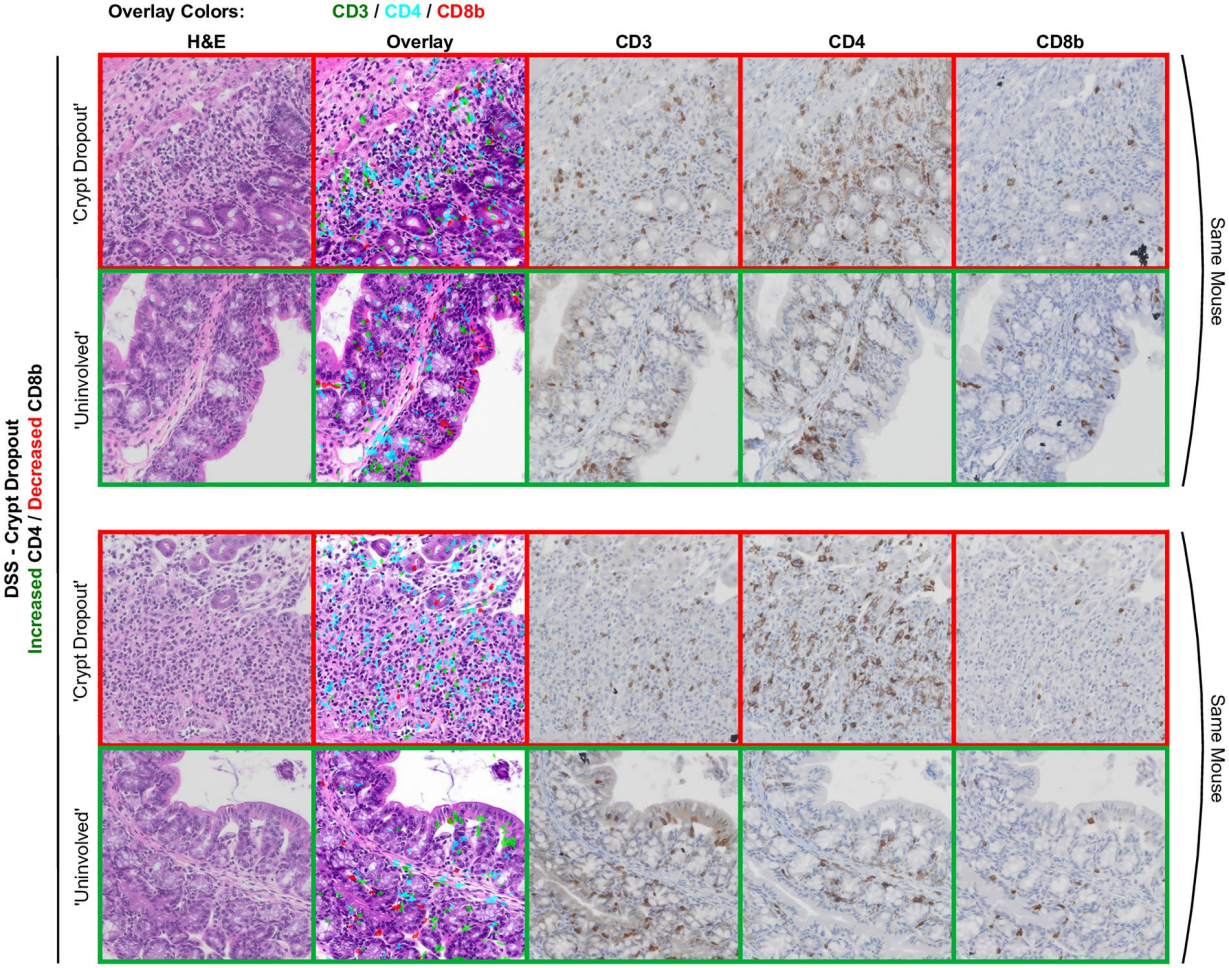
Example ‘Crypt Dropout patches from DSS-treated mice. An H&E-stained, “pseudo-multiplex” IHC, CD3-stained, CD4-stained, and CD8b-stained patch is shown for each region. For every ‘Crypt Dropout’ patch, a paired ‘Uninvolved’ region from that same mouse is shown. Cd8b depletion is observed in DSS ‘Crypt Dropout’ patches.

**Figure 9:**
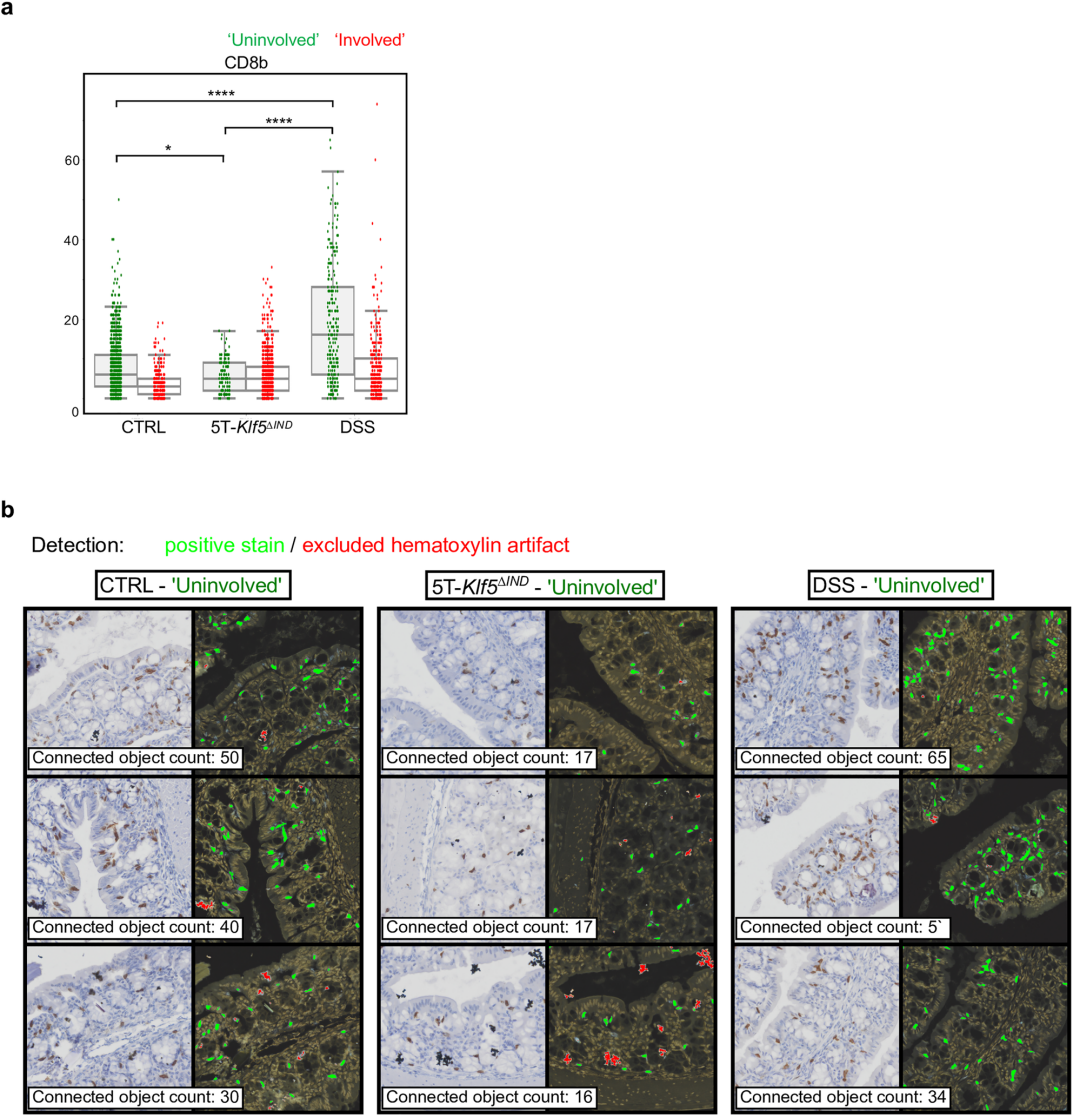
DSS-treated mice show enrichment of CD8b staining in ‘Uninvolved’ regions. (**a**) Same IHC connected object count data as Fig. 7 but with student’s t test between ‘Uninvolved’ patches across mouse conditions using *p < 0.05, **p < 0.01, ***p < 0.001, ****p < 0.0001. (**b**) Example patches of CD8b-stained ‘Uninvolved’ patches showing higher marker positivity and intraepithelial layer residence in DSS-treated mice. Each patch is taken from a different mouse. Corresponding IHC detection overlays are provided for each patch. Green refers to detected IHC positivity, while red refers to excluded hematoxylin crystal artifacts according to the method in Supplementary Fig. 4.

Possibly in line with this, ‘Crypt Dropout’ patches, the other patch class for which DSS-treated mice were enriched (Supplementary Table 1)^20^, show a similar distribution of normalized IHC marker positivity to the DSS ‘Crypt Dilation’ class (Fig. 6d). Extraction and examination of pseudo-multiplex IHC and corresponding patches also show enrichment of CD4 with a decrease in CD8b positivity relative to ‘Uninvolved’ regions in DSS-treated mice (Fig. 8). The recruitment of CD4-expressing myeloid populations likely explains the CD4 positivity, as CD4 expression has been observed in macrophages and dendritic cells^44,45^. Compared to the other IHC markers, CD4-expressing cells have morphological heterogeneity, with some cells appearing wispier (Figs. 1b, 7, 8 and Supplementary Fig. 5). Future work will evaluate whether the immune responses in expanded spaces between DSS' Crypt Dilation' patch glands are analogous to that observed in the 'Crypt Dropout' classes. Additionally, CD8b positivity is greater in ‘Uninvolved’ regions of DSS-treated mice relative to 5T-*Klf5*^*∆IND*^ mice or controls (Fig. 9a). Qualitatively, many of these CD8b + populations reside in the intraepithelial layer (Fig. 9b). This spatial-aware approach has shown that the DSS model, while believed to be myeloid-mediated, may be valuable in studying protective CD8b+ populations. As a future direction, CD8b+ populations will be characterized within these ‘Uninvolved’ regions to assess whether they have protective functions.

## Discussion

IHC staining has been used to explore the role of CD3+ , CD4+ , and CD8+ T cells in existing colitis research^46–50^. However, our method is the first to spatially map computationally detected IHC marker positivity to deep learning-generated H&E histological phenotypes of murine colitis. Furthermore, we leverage computational approaches to extend analysis across multiple mouse models concurrently. To focus on the development of this methodology, we opted for IHC targets that have already been utilized in existing literature. We plan to introduce functional markers in future work, especially considering the growing application of single cell studies to mouse models of colitis^51–53^.

Even with the relatively simple panels and IHC detection approach, shifts in immune marker prevalence are captured. For example, CD8b positivity was depleted in DSS-treated mice but not in 5T-*Klf5*^*∆IND*^ mice. While some have reported an increase in CD8+ T cells in the DSS model^54,55^, others have reported a decrease^49^ or lack of change^56^. Some have reported that CD8 depletion exacerbated DSS-induced colitis^57^, while others have reported no difference^58^. In addition to changes in institutional environments or DSS percentages, part of this variability is likely due to the mixed analysis of regions involved and uninvolved with disease and the variable proportion of diseased regions based on induction protocol. Studies typically compare immune findings between a mouse model and control but not between involved and uninvolved regions within single murine colons. A computational histological pipeline thus has the potential of normalizing and controlling immune characterization studies by the proportion of histological colonic disease on a per-mouse basis and by mapping markers to diseased regions. Given that CD8 + T cells have been reported to produce IL-17A in IBD^59,60^, a future direction will be to investigate whether the CD8b+ T cells in the 5T-*Klf5*^*∆IND*^ ‘Crypt Dilation’ patches are secreting the cytokine. Additionally, while CD8b was depleted in regions with disease in DSS mice, marker positivity was higher in ‘Uninvolved’ regions relative to 5T-*Klf5*^*∆IND*^ mice. Future directions will also involve further phenotyping of these CD8b+ populations in DSS mice to assess protective functionalities, including memory and resident population markers.

One central area for improvement of our current approach is in IHC detection. Here, an image processing approach that is simpler but more easily expanded for various IHC markers was adopted. A drawback is the inability to deal with clumped cells. Since the approach relies on the thresholded detection maps, clumped positivity will be interpreted as one connected object count. However, the current focus is on the significant effects of IHC positivity on H&E-based glandular phenotypes. This approach still captures these more significant fold-change shifts and operates as a hypothesis generator for future studies with more precise IHC detection and inclusion of more specific markers. As a future direction, the tissue detection maps with some manual annotations to indicate cell boundaries can be leveraged to train instance-level segmentation models, such as U-Net^61^. In addition, multiplex IHC is a powerful method for histological immunophenotyping through the simultaneous staining of multiple targets^62,63^. We also plan to incorporate multiplex IHC detection in future work.

Despite these limitations, our results provide the foundations and beginnings for a computational pipeline to map IHC positivity to H&E-based histological phenotypes. The great motivation is to incorporate multiple mouse models better to simulate patient phenotype heterogeneity on the histology level. Histology captures a visual snapshot in time of the cascade of events that have occurred to that point, yet histological phenotypes remain underutilized in colitis mouse model characterizations. Provided the clinical heterogeneity of IBD, individual patients are likely more represented by specific mouse models than others, and multi-mouse model experiments may thus better simulate clinical heterogeneity. Relatedly, we have shown previously that the 5T-*Klf5*^*∆IND*^, DSS-treated, and a third model with a combined induction have differential collections of H&E-detected histological abnormalities that have also been reported in patients^20^. Importantly, human IBD specimens are collected at across various timepoints, dependent on factors such as time since onset, active or remission phases, and treatment modalities. Mouse models as a collection offer an exciting avenue to model this clinical heterogeneity. We hope to further expand to add chronic and recovery period specimens, such as the chronic DSS model with water recovery periods interspersed between periods of induction^12^, to allow sample collection across various points in the treatment schedule. Furthermore, we aspire to study the impact of biologic therapies, such as anti-IL12/23, anti-TNFα, or anti-IL17, on the histopathological features in mouse models. It will also be of interest to assess the histological phenotypes of responders and non-responders across mouse models. As histological abnormalities likely reflect the molecular and immune changes occurring during disease processes, histological phenotypes may serve as compelling visual landmarks to generate mouse model-grounded characterizations of clinical patients. To this end, we plan to develop approaches to survey murine-defined H&E histological phenotypes in clinical specimens.

While the colitis mouse models included here are insufficient to capture patient heterogeneity fully, they display variable histological findings from each other. Therefore, they present a relevant data source to explore the potential for computational approaches in detecting and deriving quantifiable histological phenotypes across colitis mouse models. Our initial results also support that these phenotypes can be linked to IHC positivity to provide immune descriptions. We plan to build upon these initial methods to further characterize and develop immunologically-ground histological phenotypes across multiple colitis mouse models.

## Methods

All methods stated herein were performed in accordance with the relevant guidelines and regulations.

### Mouse cohort

The sources of *Villin-CreER*^*T2*^ (*Klf5*^*Ctrl*^) and *Villin-CreER*^*T2*^*; Klf5*^*fl/fl*^ (*Klf5*^*ΔIND*^) mice on a C57BL/6 background were previously described^27^ and bred in house. All mouse studies were approved by the Stony Brook University IACUC and were conducted in accordance with the ARRIVE guidelines (https://arriveguidelines.org). Female mice aged between 8 and 12 weeks were used in the report. Daily observations of body weight and abnormalities, including anorexia, rectal prolapse, intractable diarrhea, ruffled fur, labored breathing, hunched posture, or normal investigative behavior, were performed to minimize distress. If any mouse exhibited these observations or lost greater than 15% of baseline weight, they were euthanized immediately. Euthanasia was performed by delivering carbon dioxide via compressed gas, followed by immediate cervical dislocation. Total mouse numbers across genotypes and treatments are available in Table 1.

Mouse treatment schedules are available in Supplementary Fig. 1b. For the 5T-*Klf5*^*∆IND*^ model, tamoxifen is injected for five days intraperitoneally (IP) for an inducible intestinal epithelium-specific knockout of *Klf5* to cause colitis. Given that tamoxifen must be dissolved in corn oil before induction, *Klf5*^*∆IND*^ mice are injected for five days with IP corn oil as a control (5C-*Klf5*^*∆IND*^). In the DSS model, DSS is administered to mice by dissolving in drinking water for seven days to cause acute colitis. For the experiments in this study, 3% DSS is utilized. Of note, one archived specimen collected by past lab members is included. This mouse received 5 days of IP corn oil to control for injections in the 5T-*Klf5*^*∆IND*^ mouse model and is designated as 5C-*Klf5*^*∆IND/*+^ + DSS (Supplementary Fig. 1b, Table 1). When collected at a different institution, this mouse received 2.5% DSS, the optimized concentration. Otherwise, DSS-treated mice receive no injections, due to the previous observation that DSS-induced histology was not dependent on the corn oil injections^20^ and are designated *Klf5*^*WT*^ + DSS mice (Supplementary Fig. 1b, Table 1).

### FFPE stack generation

Mouse colons were collected and Swiss-rolled as previously described^39^. They were submitted to the Stony Brook Research Histology core for FFPE and sectioning at 5 microns per slide. For every mouse, FFPE sections were collected with their order logged. As such, slides are always stained in the same order regarding H&E and IHC markers (Fig. 1a,b). The Stony Brook Histology core stained all the slides at position 1 with hematoxylin and eosin (H&E).

### IHC staining

FFPE sections were rehydrated with xylene and 100%/95%/70% ethanol gradients. After washing in water, sections were pressure cooked at 110 °C for 20 min. Blocking was performed for 1 h at 37 °C with 5% BSA and 10% normal goat serum in 1× TBST. The primary antibody incubations were performed overnight at 4 °C on an orbital shaker. Primary antibody was diluted in 2.5% BSA and 10% normal goat serum in 1× TBST. Endogenous peroxidase activity was blocked the following day with 30 min of incubation in 2% hydrogen peroxide in methanol at room temperature. Secondary antibodies incubations were performed for 30 min at 37 °C with antibody diluted in 2.5% BSA and 10% normal goat serum in 1× TBST. Slides were then developed according to manufacturer instructions (Biocare Medical Betazoid DAB Chromogen Kit, BDB2004). Slides were counterstained with 45 s of hematoxylin and ten dips in lithium carbonate. After dehydration with 70%/95%/100% ethanol gradients and xylene, slides were mounted using a xylene-based mounting media.

### WSI generation

The H&E- and IHC-stained glass slides were scanned at 40× (0.17 µM/pixel) and digitized to a .vsi format by the Olympus VS120 Digital Virtual Slide System (VS120-L100-W). The files were then converted to a multiresolution tiff format using the quip_converter repository maintained by the Stony Brook Department of Biomedical Informatics ^64^.

The WSIs are first downsized by a factor of 2, as this resolution is utilized for WSI registration and IHC marker positivity detection, which are covered below. Then, these registered, downsized WSIs are scaled again by a factor of 4 for a total downsized factor of 8. This further downsampling is required for extracted H&E patches to be at the correct resolution to match requirements for the ‘Involved’ versus ‘Uninvolved’ classifier pipeline.

### Initial WSI scaling and computational registration of serial sections

WSIs are scaled down by a factor of 2 from the original input. They are then uploaded onto the QuIP system maintained by the Stony Brook Department of Biomedical Informatics^41^. As the WSIs contain two swiss rolls per image, individual swiss rolls are manually annotated from each H&E- and IHC-stained slide. Swiss rolls are extracted from manual annotation polygon coordinates, then placed onto a rectangular, white background. Importantly, the size of this white background is calculated to have a modulus of 0 when dividing either dimension by 4. This helps ensure an absence of rounding errors when scaling down by an additional factor of 4 with no rounding errors in the H&E characterizations.

These extracted swiss rolls are then computationally registered via a deformable method WSIReg2d^40^. Since these are stacks of serial sections, computational registration is performed iteratively. First, the second section is registered to the first. Then, the third section is registered to the already registered second section. To qualitatively evaluate registration quality, overlays are generated for each registered section. In each overlay, the fixed tissue section is the base H&E-stained WSI. For each serially-sectioned, IHC-stained WSI, a tissue map of the corresponding tissue section is overlayed in white over the base H&E. Proper overlap between the white tissue mask and base H&E indicates adequate registration quality (Fig. 2b). For the final IHC detection analysis, only one swiss roll is included for each sample.

### Characterization of H&E Slides

The H&E-stained slides have been scaled down by a factor of 2 and registered onto the same XY coordinate system as the other slides. For H&E characterizations using the ‘Involved’ versus ‘Uninvolved’ pipeline, the H&E-stained swiss rolls are scaled down further by a factor of 4 for a total downsample factor of 8. This matched the resolution the ‘Involved’ versus ‘Uninvolved’ Classifier required. H&E characterizations were then performed using the ‘Involved’ versus ‘Uninvolved’ Classifier as previously described^20^. A brief overview is provided here.

First, 32 × 32 pixel patches are extracted from H&E-stained WSIs, and the Small Patch Classifier is applied to output ‘Background’, ‘Muscle’, ‘Tissue’, and ‘Submucosa’ predictions. Tissue maps are generated with regions corresponding to informative (‘Tissue’ and ‘Submucosa’) or non-informative (‘Background’ and ‘Muscle’) regions. For every subsequently extracted 224 × 224 pixel for analysis, the corresponding region from the tissue maps are inspected. Patches are kept if less than 65% of the patch is deemed non-informative. Patches are initially extracted by regularly gridding H&E-stained WSIs by patch coordinates. Then, initially kept H&E-stained patches serve as landmarks to direct extraction of overlapping patches. Next, overlapping patches were extracted by taking initial patch coordinates and iterating 20 pixels 10 times in each direction (up/down/left/right). Finally, only informative patches are again kept.

The ‘Involved’ versus ‘Uninvolved’ Classifier is applied, and outputs generate two new datasets—one with all ‘Involved’ patches across the dataset and one with all ‘Uninvolved’ patches. The ‘Involved’ versus ‘Uninvolved’ Classifier is used as an encoder to convert all ‘Involved’ image patches into 512-length numerical vectors. These numerical representations encode high-level features that our final RN-34 classification layer evaluates and finds informative during ‘Involved’ versus ‘Uninvolved’ classifications. As a result, clustering on these representations may reveal data patterns, or image patch classes, within these binary classes. The pipeline performs principal component analysis (PCA)-based dimensionality reduction and then applies the previously published k-means model to designate each ‘Involved’ patch class into one of the ‘Involved’ or ‘Uninvolved’ H&E patch classes. Finally, as an internal control, the pipeline predicts the mouse model using the ‘Uninvolved’ patch and ‘Involved’ k-means patch class proportions to ensure the trained linear determinant analysis (LDA) classifier predicts the correct mouse condition.

### Detection of IHC marker positivity

The Scikit-Image function rgb2hed^42^ is utilized for IHC detection. The function takes in an IHC-stained patch and deconvolutes into hematoxylin and DAB channels. Using the public Pillow library^43^, the DAB channel output for each IHC-stained patch is converted to grayscale and contrast-enhanced. This output is then thresholded by intensity to generate a positive detection mask. Connected objects below or above defined size thresholds are eliminated. The input RGB patches are color inverted and then separated by intensity thresholding to exclude dark hematoxylin crystal artifacts from quantification (Supplementary Fig. 4). Intensity thresholds, contrast enhancement scales, and minimum and maximum size cutoffs are defined and optimized for each marker separately, as values can depend upon factors like the morphology of stained cells. For the final quantification of marker positivity, connected objects are counted. While this approach cannot adequately separate clumped cells, the connected counts rely on capturing areas of high and low marker positivity.

### “Pseudo-multiplex IHC” generation” and IHC mapping

Computational registration is performed at the same resolution (downsample factor of 2) for both H&E- and IHC-stained slides. As such, IHC marker positivity detection maps can be directly overlayed back onto H&E WSIs using patch XY coordinates. Overlayed IHC positivity is provided in different colors based on the IHC marker.

For IHC-HE mapping, the H&E ‘Involved’ versus ‘Uninvolved’ outputs occur at a lower resolution (downsample factor of 8). The cropped Swiss rolls from QuIP extraction were pasted onto white background images with dimensions causing no rounding errors when resizing between scales to allow the joining of outputs taken from different downsampling factors. As a result, the XY coordinates of H&E ‘Involved’ versus ‘Uninvolved’ patch outputs need to be multiplied by 4 to match up with IHC detection outputs. Two additional preprocessing steps are taken when mapping to H&E ‘Involved’ k-means patch classes. First, the ‘Involved’ region IHC marker positivity is normalized within each mouse to average ‘Uninvolved’ region counts. Specifically, the ‘Uninvolved’ patches for each mouse are first gathered. For each IHC target, marker positivity is averaged across all of one mouse’s ‘Uninvolved’ patches. The ‘Involved’ patches are then iterated, and IHC marker positivity is normalized to the average ‘Uninvolved’ patch count of that IHC marker for that mouse. This approach allows the identification of IHC markers preferentially enriched in ‘Involved’ relative to ‘Uninvolved’ regions within each mouse. The second preprocessing step focuses on ‘Involved’ patch classes with at least 10% frequency per mouse model. Each patch class was quantified across mouse models and provided a weight of 0 if below 10% frequency. While a future direction is incorporating weighting based on patch frequency, the focus was placed here on the dominant histological patterns for each mouse model. This permitted the exploration of the potential and viability of defining IHC-H&E histological phenotypes.

### Supplementary Information

Supplementary Legends.

Supplementary Figures.

Supplementary Information 1.

Supplementary Table 1.

Supplementary Table 2.

## Data Availability

H&E- and IHC-stained WSIs are available at the following links: https://datadryad.org/stash/landing/show?id=doi%3A10.5061%2Fdryad.qz612jmms (H&E), https://datadryad.org/stash/landing/show?id=doi%3A10.5061%2Fdryad.ffbg79d0q (IHC_Part 1), and https://datadryad.org/stash/landing/show?id=doi%3A10.5061%2Fdryad.wm37pvmt0 (IHC_Part 2). Code will be made available at https://github.com/skobayashi0417/IHC_HE_pipeline.git.
