# Supplementary material for: Computational immunohistochemical mapping adds immune context to histological phenotypes in mouse models of colitis
Source: Sci Rep. 2023 Sep 1;13:14386. doi: 10.1038/s41598-023-41574-8 (PMC10474139; doi:10.1038/s41598-023-41574-8)
Supplement: Supplementary file 1 — Supplementary Legends. [file 41598_2023_41574_MOESM1_ESM.docx]

**Supplementary Figure and Table Legends**

**Supplementary Figure 1. General sample collection approach and mouse model treatment schedules.** A) Colitis and control mice are treated. Colons are harvested, then formalin-fixed and paraffin-embedded (FFPE). Serial sections are taken from FFPE colons with logged order of slides. Slides are stained for hematoxylin and eosin (H&E) or immunohistochemistry (IHC) in the same order for all samples, then digitized by a whole slide scanner to generate whole slide images (WSIs). B) mouse model treatment schedules.

**Supplementary Figure 2. Small Patch Classifier and patch filtering overview.** A) For each input H&E-stained WSI, a tissue map is generated using the predictions from the Small Patch Classifier [20]. B) Example 224x224 pixel H&E-stained patches with corresponding patches extracted from tissue maps. For each extracted patch, filtering decision is made based on whether there is more than 65% (filter) or less than 65% (keep) of unwanted Background/Muscle area on the corresponding tissue map patch. Figures were adapted from Kobayashi et al. *PLOS ONE* (2022) [20].

**Supplementary Figure 3. ‘Involved’ and ‘Uninvolved’ patch proportions by mouse.** Proportions of ‘Involved’ and ‘Uninvolved’ patch classes for individual mice corresponding to Figure 3B are shown. Specific genotypes and treatments for each mouse are available in Supplementary Table 2.

**Supplementary Figure 4. Overview of IHC detection approach.** For each IHC-stained patch, the DAB channel is extracted using the SciKit Image rgb2hed function [42] and intensity thresholded. The IHC patch is also color inverted before another intensity thresholding to detect hematoxylin crystal artifacts. Outputs of the two are merged so that final IHC connected object counts include any hematoxylin crystal false positives.

**Supplementary Figure 5. IHC detection connected object counts capture shifts in marker positivity.** For each IHC marker, example paired IHC-stained patches and IHC detection outputs are shown for regions with high, medium, and low staining across 5T-*Klf5^∆IND^*^,^ DSS-treated, and control mice. High, medium, and low patch examples for a certain IHC target and mouse condition are taken from the same sample. In IHC detection overlays, green refers to detected IHC positivity, while red refers to excluded hematoxylin crystal artifacts according to the method in Supplementary Figure 4.

**Supplementary Figure 6. Per mouse ‘Involved’ and ‘Uninvolved’ IHC connected object counts.** Per-patch IHC connected object counts for CD3, CD4, and CD8b are shown for individual mice corresponding to Figure 5. Specific genotypes and treatments for each mouse are available in Supplementary Table 2. Student’s t-test was performed between ‘Uninvolved’ and ‘Involved’ patches for each mouse with *p<0.05, **p<0.01, ***p<0.001, ****p<0.0001.

**Supplementary Figure 7. ‘Involved’ and ‘Uninvolved’ patch class proportions per mouse.** Patch class proportions are shown for individual mice corresponding to Figure 6b. Specific genotypes and treatments for each mouse are available in Supplementary Table 2.

**Supplementary Table 1. ‘Involved’ patch class proportions by mouse conditions.** For each mouse condition, all ‘Involved’ patches were gathered, and the proportion of each ‘Involved’ patch class was calculated. Numbers marked with asterisks refer to ‘Involved’ k-means patch classes present in less than 10% frequency out of all ‘Involved’ patches. These classes were excluded from IHC mapping to ‘Involved’ k-means patch classes.

**Supplementary Table 2. Patch properties per mouse.** For individual mice, treatment and genotype, along with information regarding numbers of patches, proportions of patches, and average tissue area as estimated during Small Patch Classifier filtering (Supplemental Figure 2), are displayed.
