# Supplementary material for: Computational immunohistochemical mapping adds immune context to histological phenotypes in mouse models of colitis
Source: Sci Rep. 2023 Sep 1;13:14386. doi: 10.1038/s41598-023-41574-8 (PMC10474139; doi:10.1038/s41598-023-41574-8)
Supplement: Supplementary file 2 — Supplementary Figures. [file 41598_2023_41574_MOESM2_ESM.pdf]

Computational immunohistochemical mapping adds immune context to histological phenotypes in mouse models of colitis

Soma Kobayashi[1], Christopher Sullivan[2], Agnieszka B. Bialkowska[2], Joel H. Saltz[1,3], and Vincent W. Yang*[,1,2,4]

[1]Department of Biomedical Informatics, Renaissance School of Medicine at Stony Brook University, Stony Brook, NY, United States.

[2]Department of Medicine, Renaissance School of Medicine at Stony Brook University, Stony Brook, NY, United States.

[3]Department of Pathology, Renaissance School of Medicine at Stony Brook University, Stony Brook, NY, United States.

[4]Department of Physiology and Biophysics, Renaissance School of Medicine at Stony Brook University, Stony Brook, NY, United States.

*Corresponding Author: Vincent.Yang@stonybrookmedicine.edu (VY)

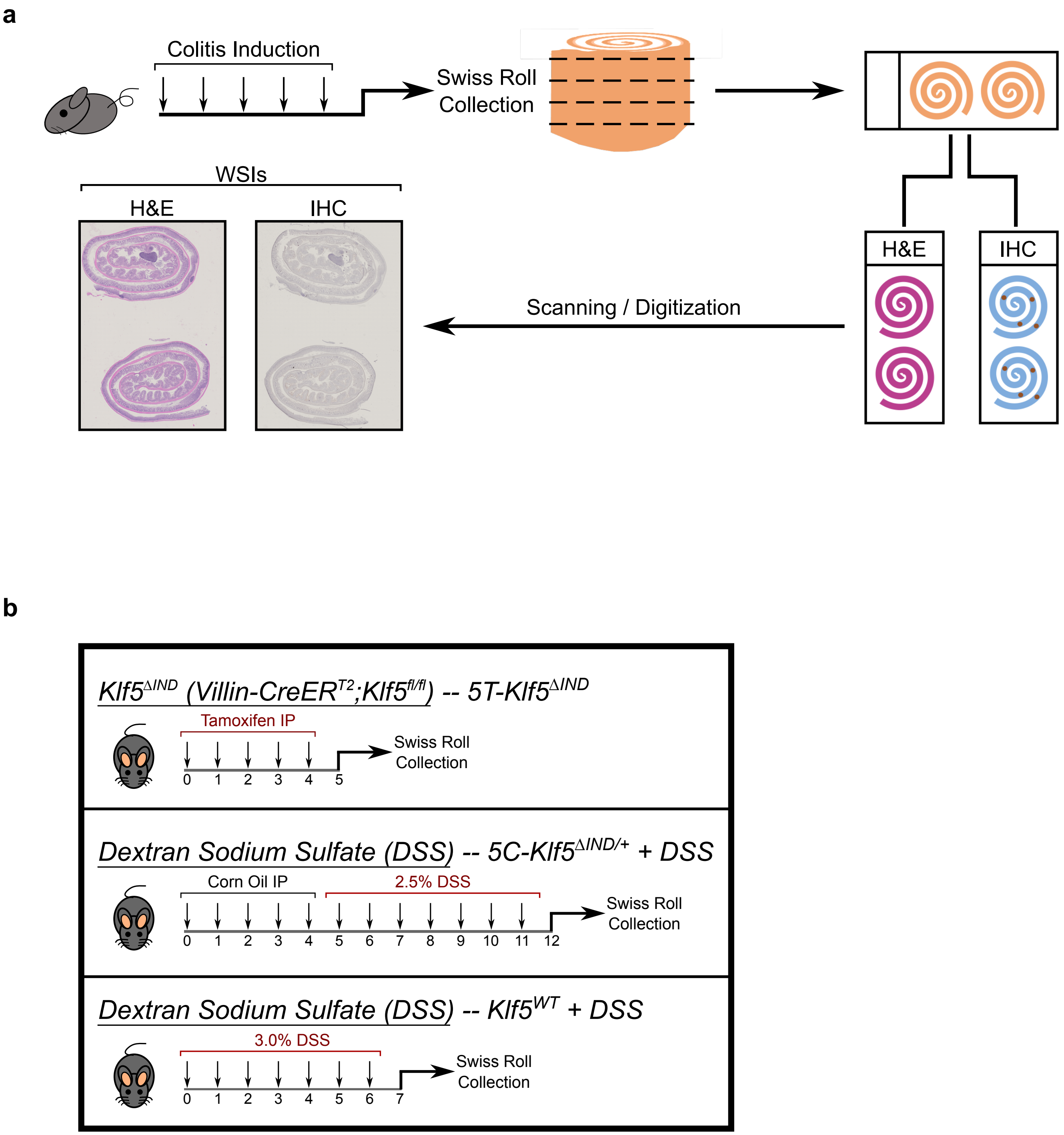

S1Fig. General sample collection approach and mouse model treatment schedules.

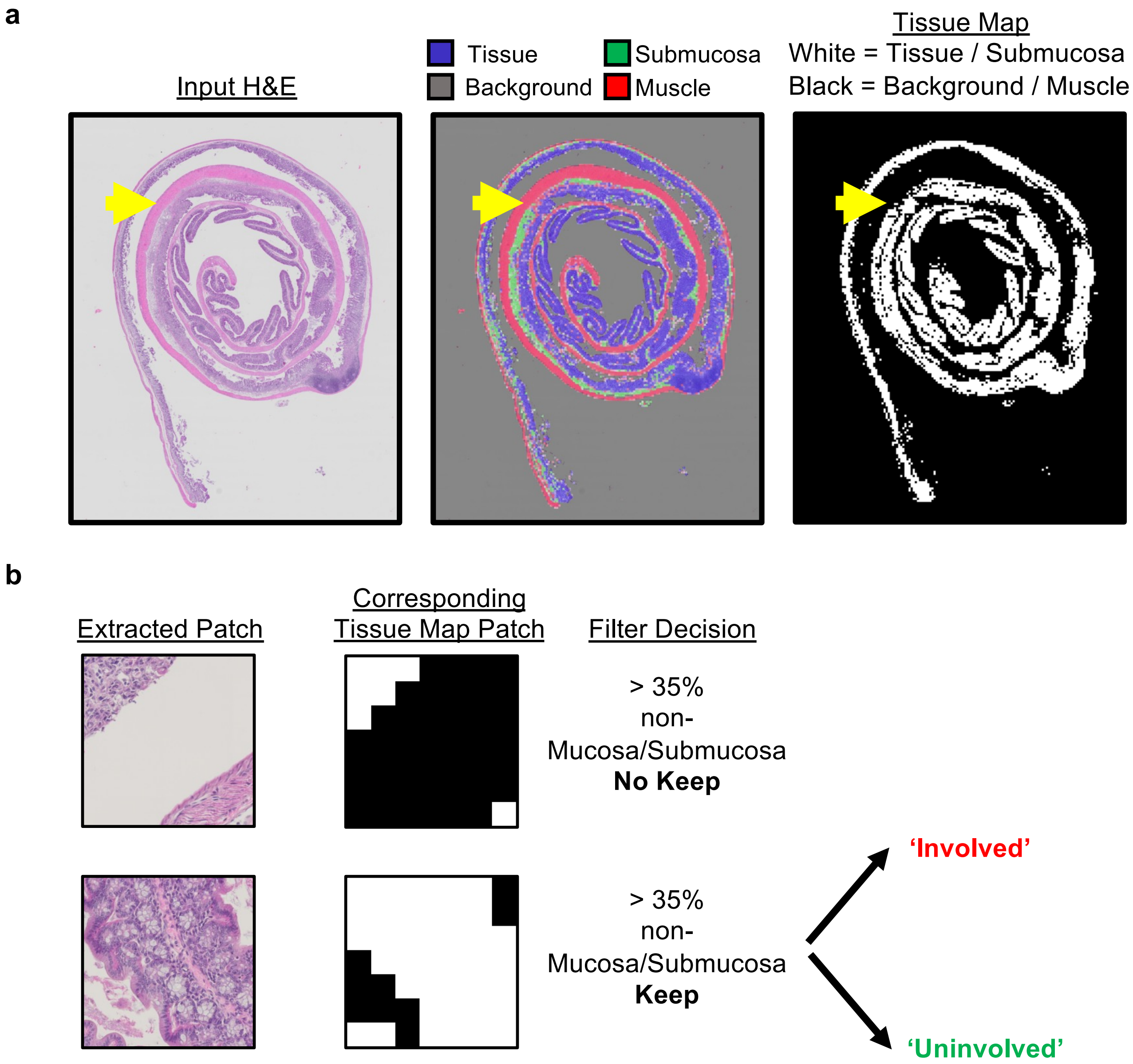

S2Fig. Small Patch Classifier and patch filtering overview.

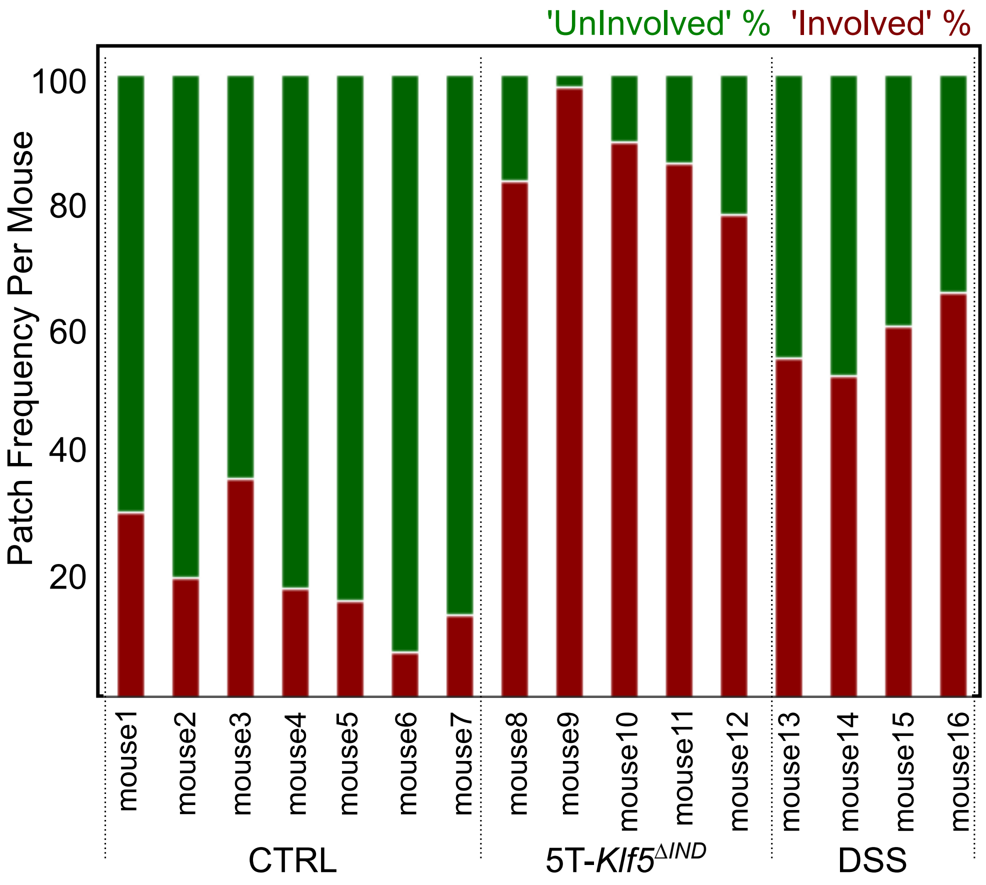

S3Fig. 'Involved' and 'Uninvolved' patch proportions by mouse.

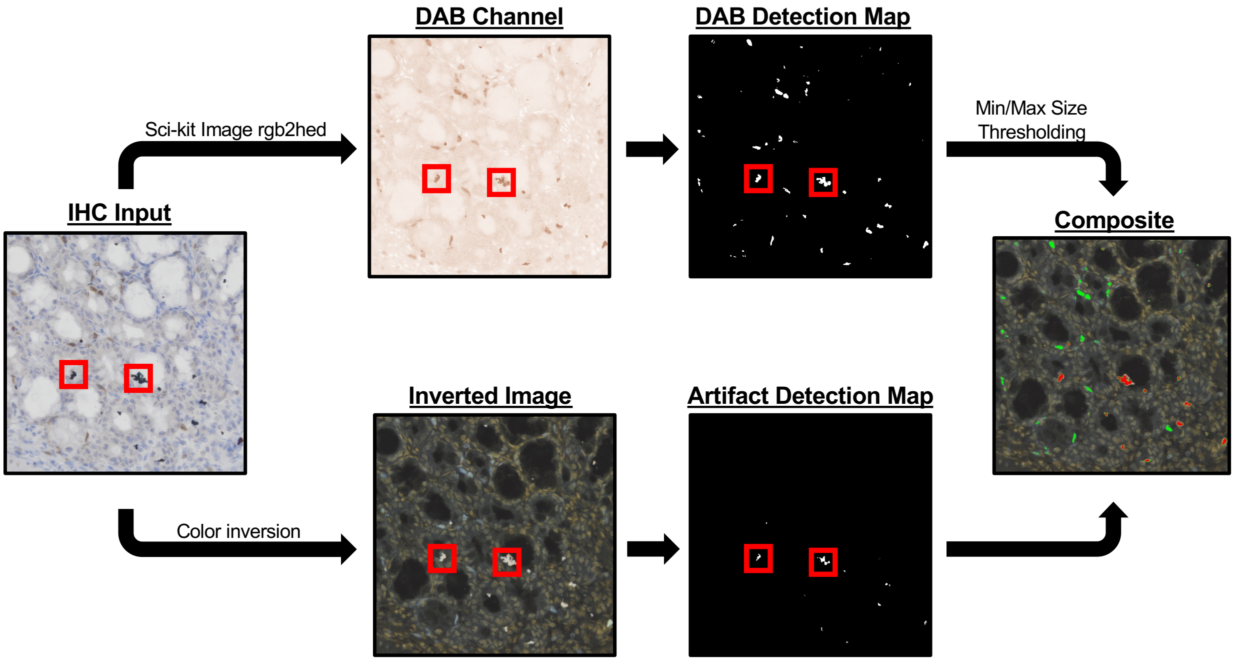

S4Fig. Overview of IHC detection approach.

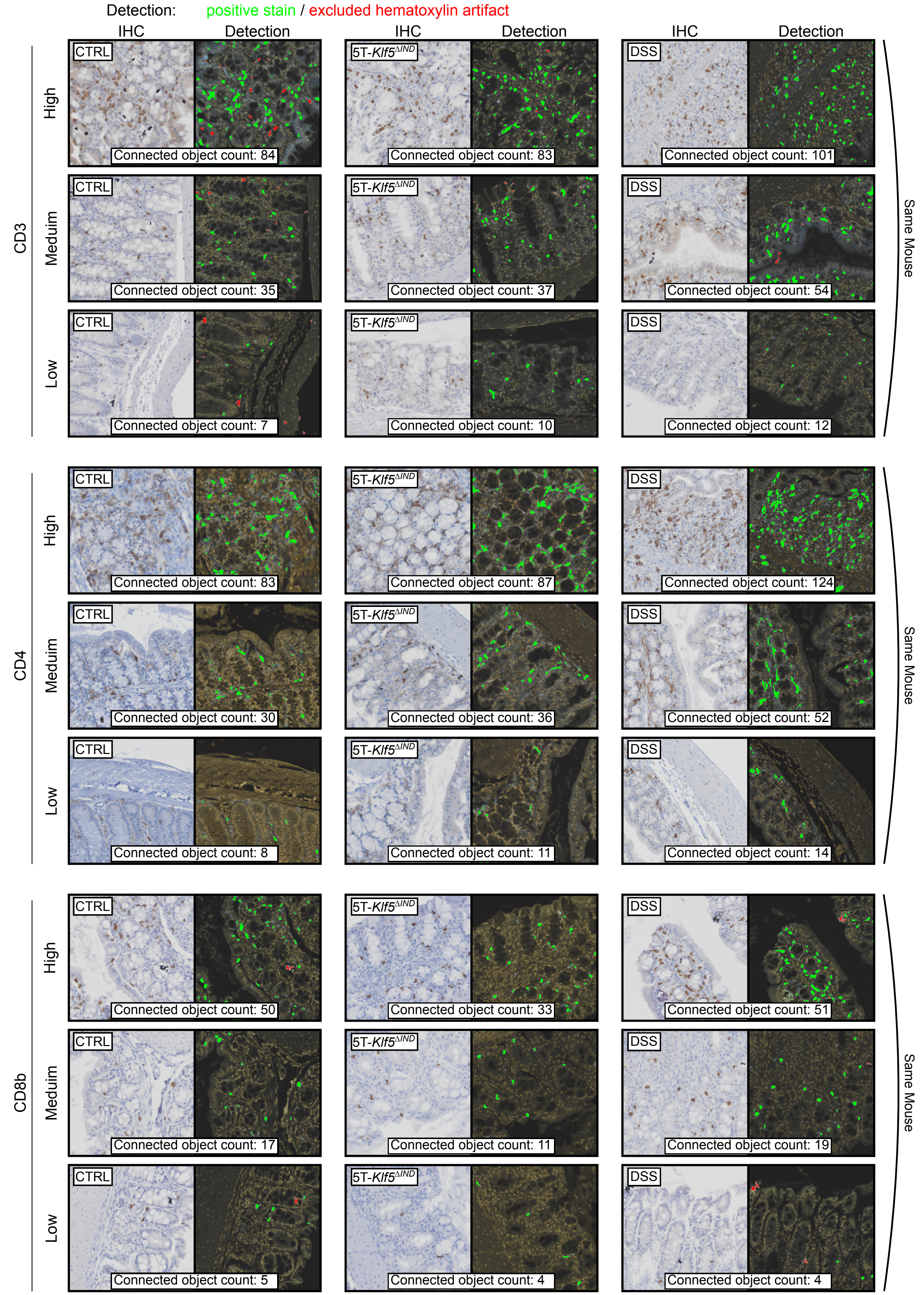

S5 Fig. IHC detection connected object counts capture shifts in marker positivity.

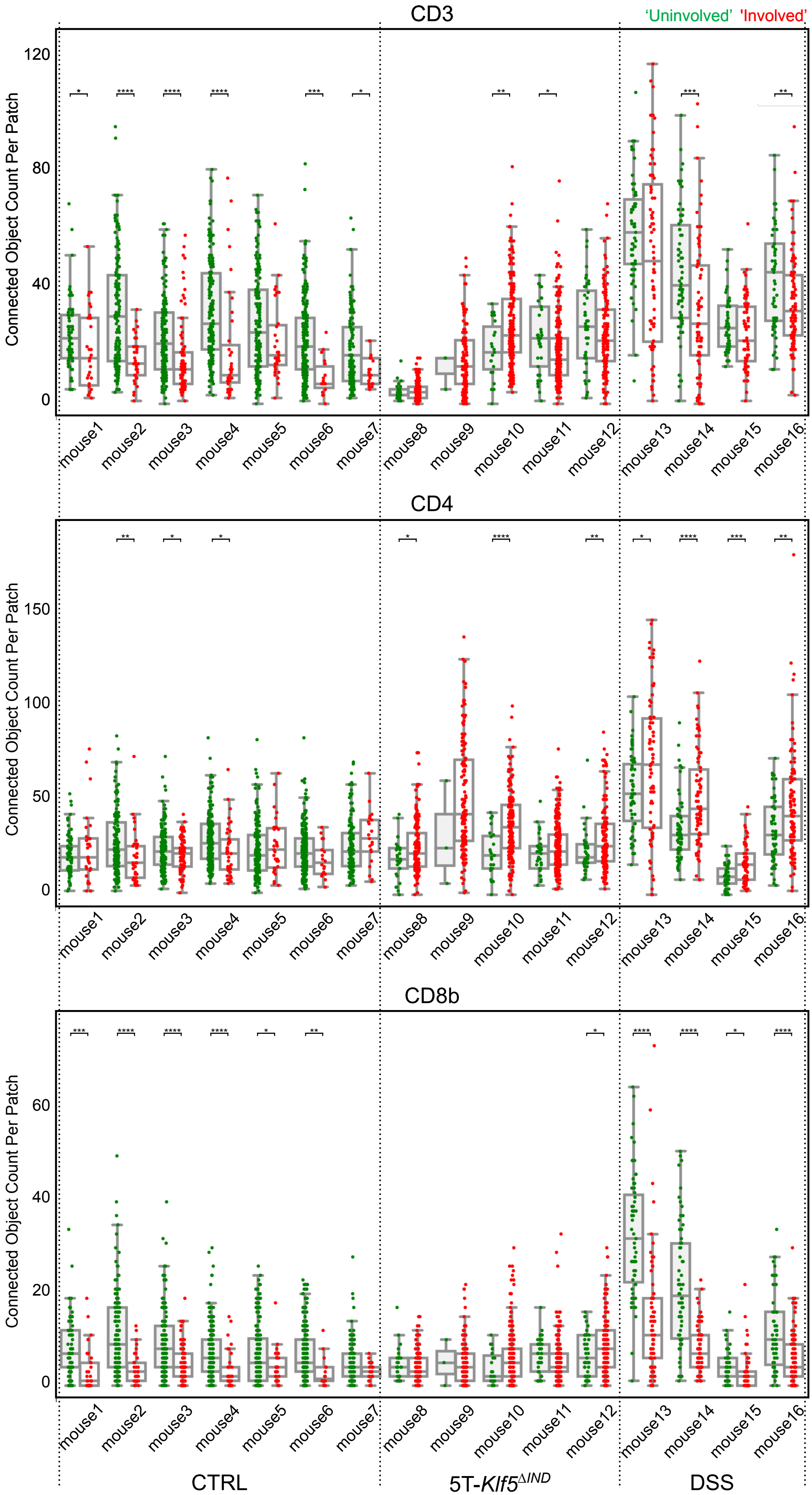

S6Fig. Per mouse 'Involved' and 'Uninvolved' IHC connected object counts.

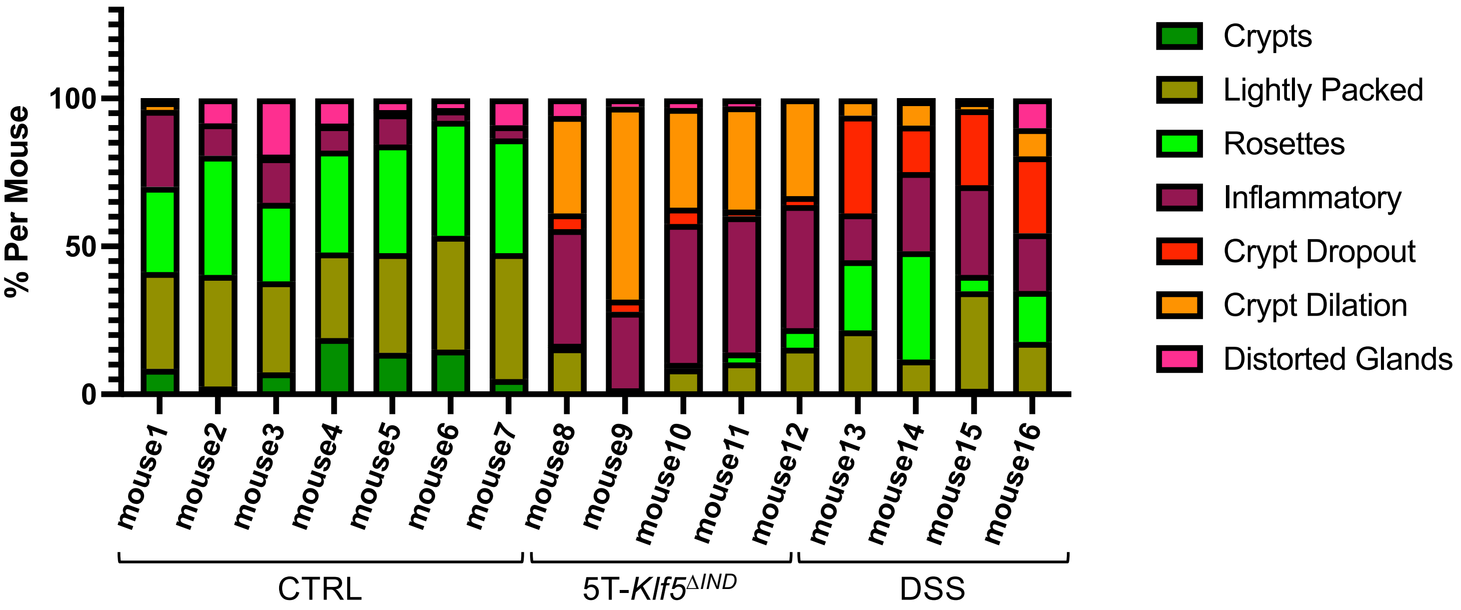

S7Fig.'Involved' and 'Uninvolved' patch class proportions per mouse.
