# Supplementary material for: Computational immunohistochemical mapping adds immune context to histological phenotypes in mouse models of colitis
Source: Sci Rep. 2023 Sep 1;13:14386. doi: 10.1038/s41598-023-41574-8 (PMC10474139; doi:10.1038/s41598-023-41574-8)
Supplement: Supplementary file 3 — Supplementary Information 1. [file 41598_2023_41574_MOESM3_ESM.docx]

**SUPPLEMENTARY METHODS**

All supplementary methods were performed in accordance with the relevant guidelines and regulations.

***IHC Patch Extraction from H&E Patch Coordinates***

After the H&E patch extraction, kept H&E patch coordinates can be used to guide the IHC patch extraction process. While IHC-stained WSIs are at a higher resolution than the H&E-stained WSIs (downsample factor of 2 versus 4), the patch extraction should similarly exclude those with too much non-informative area. Since all WSIs were initially cropped to ensure no rounding errors when moving between our scale factors (downsample factor of 2 versus 4), the coordinates of the kept H&E-stained patches can be used to direct IHC patch extraction. Specifically, H&E patch coordinates are scaled back up by a factor of 4, and then used to extract patches from IHC-stained, registered WSIs. As a result, the same numbers of patches are always extracted for every serially-sectioned WSIs from a mouse, whether they are stained for H&E or IHC.

***H&E Overlapping Patch Extraction***

Overlapping patches were extracted by taking initial patch coordinates and iterating 20 pixels 10 times in each direction (up/down/left/right). Small Patch Classifier patch filtering is repeated at every step to ensure only informative patches are kept. The ‘Involved’ versus ‘Uninvolved’ classifier is applied to each overlapping patch, and prediction confidences are averaged at every pixel in the WSI. The overlapping patches ensure that the prediction associated with each pixel considers more spatial context than just the initial patch it resided in.
