# Supplementary material for: Computational immunohistochemical mapping adds immune context to histological phenotypes in mouse models of colitis
Source: Sci Rep. 2023 Sep 1;13:14386. doi: 10.1038/s41598-023-41574-8 (PMC10474139; doi:10.1038/s41598-023-41574-8)
Supplement: Supplementary file 4 — Supplementary Table 1. [file 41598_2023_41574_MOESM4_ESM.docx]

**Supplementary Table 1. ‘Involved’ patch class proportions by mouse conditions**

|  | Inflammatory | Crypt Dropout | Crypt Dilatation | Distorted Glands |
| --- | --- | --- | --- | --- |
| CTRL | 53.82% | 0.00%* | 3.43%* | 42.75% |
| *5T-Klf5^ΔIND^* | 47.66% | 4.56%* | 44.51% | 3.27%* |
| DSS | 39.09% | 43.03% | 12.12% | 5.76%* |
