# Supplementary material for: Computational immunohistochemical mapping adds immune context to histological phenotypes in mouse models of colitis
Source: Sci Rep. 2023 Sep 1;13:14386. doi: 10.1038/s41598-023-41574-8 (PMC10474139; doi:10.1038/s41598-023-41574-8)
Supplement: Supplementary file 5 — Supplementary Table 2. [file 41598_2023_41574_MOESM5_ESM.docx]

**Supplemental Table 2. Patch properties per mouse.**

| Sample | Treatment/Genotype | Comparison Group | Total # Patches | Average % Tissue Area Per Patch (SEM) | # Involved (% of Total) | # Uninvolved (% of Total) | # 'Inflammatory' (% of Total) | # 'Crypt Dropout' (% of Total) | # 'Crypt Dilation' (% of Total) | # 'Distorted Glands' (% of Total) | # 'Crypts' (% of Total) | # 'Lightly Packed' (% of Total) | # 'Rosettes' (% of Total) |
| --- | --- | --- | --- | --- | --- | --- | --- | --- | --- | --- | --- | --- | --- |
| mouse1 | 5C-*Klf5^∆IND^* | CTRL | 104 | 64.4% (1.6%) | 31 (29.8%) | 73 (70.2%) | 27 (26.0%) | 0 (0%) | 3 (2.9%) | 1 (1.0%) | 9 (8.7%) | 34 (32.7%) | 30 (28.8%) |
| mouse2 | 5C-*Klf5^∆IND^* | CTRL | 191 | 61.2% (1.1%) | 37 (19.4%) | 154 (80.6%) | 21 (11.0%) | 0 (0%) | 0 (0%) | 16 (8.4%) | 5 (2.6%) | 72 (37.7%) | 77 (40.3%) |
| mouse3 | *Klf5^∆IND/+^* | CTRL | 241 | 62.5% (0.9%) | 85 (35.3%) | 156 (64.7%) | 37 (15.4%) | 0 (0%) | 2 (0.8%) | 46 (19.1%) | 18 (7.5%) | 74 (30.7%) | 64 (26.6%) |
| mouse4 | *Klf5^∆IND/+^* | CTRL | 221 | 62.9% (0.9%) | 39 (17.6%) | 182 (82.4%) | 19 (8.6%) | 0 (0%) | 1 (0.5%) | 19 (8.6%) | 42 (19.0%) | 64 (29.0%) | 76 (34.4%) |
| mouse5 | *Klf5^∆IND^* | CTRL | 199 | 65.2% (1.2%) | 31 (15.6%) | 168 (84.4%) | 21 (10.6%) | 0 (0%) | 2 (1.0%) | 8 (4.0%) | 28 (14.1%) | 67 (33.7%) | 73 (36.7%) |
| mouse6 | *Klf5^∆IND^* | CTRL | 239 | 66% (1.1%) | 18 (7.5%) | 221 (92.5%) | 9 (3.8%) | 0 (0%) | 1 (0.4%) | 8 (3.3%) | 36 (15.1%) | 92 (38.5%) | 93 (38.9%) |
| mouse7 | *Klf5^WT^* + H_2_O | CTRL | 155 | 64.4% (1.2%) | 21 (13.5%) | 134 (86.5%) | 7 (4.5%) | 0 (0%) | 0 (0%) | 14 (9.0%) | 8 (5.2%) | 66 (42.6%) | 60 (38.7%) |
| mouse8 | 5T-*Klf5^∆IND^* | TAM | 170 | 59.5% (1.1%) | 141 (82.9%) | 29 (17.1%) | 66 (38.8%) | 9 (5.3%) | 56 (32.9%) | 10 (5.9%) | 1 (0.6%) | 26 (15.3%) | 2 (1.2%) |
| mouse9 | 5T-*Klf5^∆IND^* | TAM | 147 | 69.2% (1.5%) | 144 (98.0%) | 3 (2.0%) | 38 (25.9%) | 6 (4.1%) | 96 (65.3%) | 4 (2.7%) | 0 (0%) | 2 (1.4%) | 1 (0.7%) |
| mouse10 | 5T-*Klf5^∆IND^* | TAM | 217 | 63.7% (1.0%) | 194 (89.4%) | 23 (10.6%) | 102 (47.0%) | 12 (5.5%) | 73 (33.6%) | 7 (3.2%) | 0 (0%) | 19 (8.8%) | 4 (1.8%) |
| mouse11 | 5T-*Klf5^∆IND^* | TAM | 261 | 64.9% (1.1%) | 224 (85.8%) | 37 (14.2%) | 120 (46.0%) | 6 (2.3%) | 91 (34.9%) | 7 (2.7%) | 1 (0.4%) | 27 (10.3%) | 9 (3.4%) |
| mouse12 | 5T-*Klf5^∆IND^* | TAM | 197 | 63.7% (1.2%) | 153 (77.7%) | 44 (22.3%) | 82 (41.6%) | 6 (3.0%) | 65 (33.0%) | 0 (0%) | 0 (0%) | 31 (15.7%) | 13 (6.6%) |
| mouse13 | *Klf5^WT^* + DSS | DSS | 139 | 69.4% (1.3%) | 76 (54.7%) | 63 (45.3%) | 22 (15.8%) | 46 (33.1%) | 8 (5.8%) | 0 (0%) | 1 (0.7%) | 29 (20.9%) | 33 (23.7%) |
| mouse14 | *Klf5^WT^* + DSS | DSS | 153 | 68.9% (1.5%) | 79 (51.6%) | 74 (48.4%) | 41 (26.8%) | 24 (15.7%) | 13 (8.5%) | 1 (0.7%) | 0 (0%) | 18 (11.8%) | 56 (36.6%) |
| mouse15 | 5C-*Klf5^∆IND/+^* + DSS | DSS | 109 | 57.4% (1.4%) | 65 (59.6%) | 44 (40.4%) | 33 (30.3%) | 28 (25.7%) | 3 (2.8%) | 1 (0.9%) | 2 (1.8%) | 36 (33.0%) | 6 (5.5%) |
| mouse16 | *Klf5^WT^* + DSS | DSS | 169 | 70% (1.3%) | 110 (65.1%) | 59 (34.9%) | 33 (19.5%) | 44 (26.0%) | 16 (9.5%) | 17 (10.1%) | 0 (0%) | 30 (17.8%) | 29 (17.2%) |
